# The ethical aspects of exposome research: a systematic review

**DOI:** 10.1093/exposome/osad004

**Published:** 2023-04-12

**Authors:** Caspar W. Safarlou, Karin R. Jongsma, Roel Vermeulen, Annelien L. Bredenoord

**Affiliations:** 1 Department of Global Public Health and Bioethics, Julius Center for Health Sciences and Primary Care, University Medical Center Utrecht, Utrecht, The Netherlands; 2 Department of Population Health Sciences, Utrecht University, Utrecht, The Netherlands; 3 Erasmus School of Philosophy, Erasmus University Rotterdam, Rotterdam, The Netherlands

**Keywords:** exposome, omics, personalized medicine, environmental epidemiology, ethics, systematic review

## Abstract

In recent years, exposome research has been put forward as the next frontier for the study of human health and disease. Exposome research entails the analysis of the totality of environmental exposures and their corresponding biological responses within the human body. Increasingly, this is operationalized by big-data approaches to map the effects of internal as well as external exposures using smart sensors and multiomics technologies. However, the ethical implications of exposome research are still only rarely discussed in the literature. Therefore, we conducted a systematic review of the academic literature regarding both the exposome and underlying research fields and approaches, to map the ethical aspects that are relevant to exposome research. We identify five ethical themes that are prominent in ethics discussions: the goals of exposome research, its standards, its tools, how it relates to study participants, and the consequences of its products. Furthermore, we provide a number of general principles for how future ethics research can best make use of our comprehensive overview of the ethical aspects of exposome research. Lastly, we highlight three aspects of exposome research that are most in need of ethical reflection: the actionability of its findings, the epidemiological or clinical norms applicable to exposome research, and the meaning and action–implications of bias.

## Introduction

After the completion and maturation of the Human Genome Project, it was found that genetic factors alone can account for only 10%–30% of disease risks.^1^ To fill the nongenetic gap, a large number of scientists have issued calls for a new research program to investigate and explain the rest of the (environmental) factors that contribute to the development of health and disease. This ambition resulted in the exposome research program. Subsequently, the exposome concept has been taken up and defined as the set of exposures and their biological responses that affect one’s body throughout the lifespan.^2,3^ In practice, exposome research has a relatively narrow interest in the exposome entity: because it is interested in health and disease, it focuses on ultimately identifying specifically those exposures that affect human health and disease, as opposed to any exposure that affects the body or those biological traces that can be used for forensic ends.

Now that exposome research is developing rapidly and draws on research fields and approaches that have originally yielded ethical debate, it is pertinent that we map and investigate the ethical issues that are relevant for exposome research. Because exposome research has not matured yet and much of its tools are still in the design phase, there is an opportunity for exposome researchers and ethicists to explicitly think about the way in which values are incorporated into exposome research.^4^ So far, the ethical implications of exposome research itself are rarely discussed in the literature. This means that there is a definite gap in the literature with regard to the analysis and evaluation of the ethical aspects of this novel development within the field of environmental health and the life sciences. In this article, we will bridge this gap by providing a comprehensive overview of the ethical aspects of exposome research by categorizing the various ethical aspects that are mentioned in the exposome research literature. However, due to the fact that we found no papers that substantively discuss the ethical aspect of exposome research by name, we also categorize the various ethical aspects mentioned in the literature of approaches and fields that underly exposome research but do not use the term “exposome”.

## Methodological choices

We conducted a systematic review of ethical considerations that are relevant to exposome research. We used an adapted version of the methodology developed by Strech and Sofaer.^5^ Their methodology is used to identify arguments that either support or oppose particular ideas. However, it does not attempt to assess the quality of these arguments, because it tries to enable the systematic collection and description of all the relevant articles in which particular arguments occur. We have chosen to review ethical aspects instead of “reasons for and against”, as our aim is to map the ethical aspects of exposome research and not to narrowly identify the reasons for and against doing exposome research. When identifying moral or ethical aspects (used synonymously here), we took these to be value-based considerations that (1) are relevant to exposome research and (2) are not exclusively related to natural-scientific fact finding.^6^ For example, an author can state that a new assay is valuable for understanding the function of certain metabolites. But only when an author relates the assay to other values, such as participant privacy, research standards, or public health policy, then we include their consideration in our review. We adopted the relevant elements of the Preferred Reporting Items for Systematic Reviews and Meta-Analyses (PRISMA) statement.^7^

### Search strategy

We first performed a search that included the term “exposome” or potential derivatives of the word “exposome” and ethics, morality, or words that are derivative from these (Supplementary File S1). This search yielded nine unique articles, none of which substantively addressed the ethics of exposome research. In the absence of more results, we chose to construct a search query that covers not only the term “exposome” and derivative words, but also related research fields and approaches that come together in the exposome research program, such as the various - omics fields, biomonitoring, and biomarkers research.^8^ We performed a short check for each added term to see if many new articles were added, in order to avoid the gathering of too many articles to subsequently analyze. Because we included a large amount of articles into our final analysis, we have chosen not to identify additional articles via snowballing. To improve the choice of words in our query, the choice of databases, and our usage of technical search functions, we consulted with a trained librarian and analyzed queries used by other systematic ethics reviews. We used PubMed, Scopus, and Web of Science because these databases cover published material within biomedical and epidemiological research in a comprehensive way (Supplementary File S1). We performed our original searches on February 12, 2021 and performed an update search on June 8, 2022.

### Article selection and inclusion criteria

**Figure 1:**
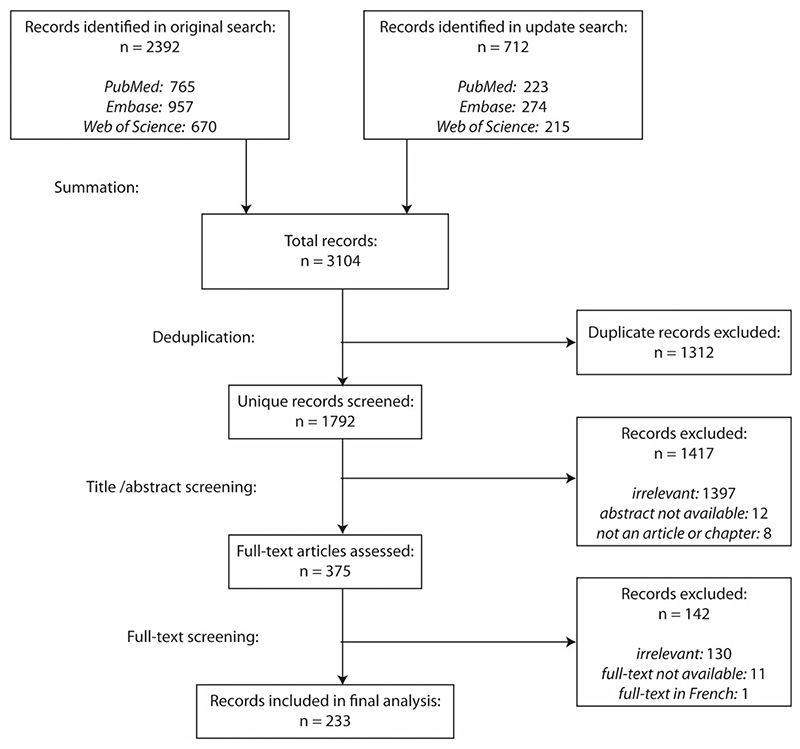
Flowchart of article screening process phases.

We excluded articles that were not written in either Dutch or English, did not have an abstract or full-text available, or either did not mention any ethical aspects or were not related to exposome research (labeled as “irrelevant”). We found no articles in Dutch. Articles from journals or book chapters were included, but conference abstracts were excluded because this article type would not provide sufficient information to describe the considered ethical claims of the authors. Publications from before the term exposome was coined were not excluded for two related reasons. First of all, because exposome research brings together many related research fields and approaches, this implies that older articles might still present ethical aspects that are relevant for exposome research today. Second, we believe that it would benefit the quality of further ethical analyses to consider the historical development of ethical considerations that have been put forward in the literature. If an article had no abstract available, then we read the first paragraph of the article to assess inclusion into the full-text analysis. If a first paragraph was not clearly delineated, then we read the first page of the article to assess inclusion into the full-text analysis. We used Rayyan software to assign exclusion criteria to papers during both the title/abstract and full-text screening. For the flowchart of the article screening process phases, see Figure 1.

### Data extraction, analysis, and synthesis

To categorize the various ethical considerations that we found, we used a thematic analysis to connect ethical aspects mainly to categories that are important from the perspective of the practice of exposome research (as opposed to particular bioethical principles). For example, we formulated “applied” themes such as “research program goals” and “research standards”, instead of creating wide themes such as “autonomy” and “beneficence”. We have done so for two reasons. First, because categorizing ethical considerations by reference to parts of exposome research immediately connects such considerations to the relevant scientific context, thereby directly showing both their importance and practical relevance. Connecting ethical considerations primarily to broad bioethical principles or general values would not immediately make clear to what aspect of exposome research they are important or relevant, and making their relevance explicit would then require an extra explanatory step. Second, we have done so because it allows exposome researchers to browse many of the various ethical aspects by referring to topics as they arise in their work, instead of finding multiple aspects of their research under the heading of principles of biomedical ethics with which they might be unfamiliar. We hope that such easy accessibility facilitates researcher engagement with the ethical aspects of their research. Nevertheless, sometimes papers do make comments related only to particular biomedical values or principles, in which case we have dedicated paragraphs on them.

With respect to the synthesis/formulation of themes, subthemes, and paragraphs, we employed a combination of inductive and deductive reasoning. For example, we used our knowledge of privacy, informed consent, the reference exposome, and expotypes to deductively infer whether a specific ethical aspect fits under one of these categories. On the inductive side, we found that certain ethical aspects necessitated the creation of categories such as study participation, the distinction between participant and patient, and exposomic actionability for individuals. Many categories were created using both inductive and deductive reasoning. The category that most explicitly uses both forms of reasoning is the section on bias in data, analysis, algorithms, and artificial intelligence (AI), as we deductively inferred that many ethical aspects discuss bias, while inductively, we subsequently discovered a need to (partly) disambiguate the term “bias” in order to clearly categorize ethical aspects.

During the full-text assessment and qualitative synthesis, we assigned broadly formulated labels to papers using Rayyan software and highlighted and commented on ethical aspects in papers using Endnote or Mendeley software. Subsequently, per label, we extracted ethical aspects from included papers into tables using Microsoft Word and (re)assigned them to (newly created) categories in our review via an iterative process. Aspects and categories were both discussed between the authors until agreement was reached on their formulation.

Within the text, we have aimed to present the ethical aspects brought forward by disparate papers in a logical order. Both sections and paragraph breaks are intended to convey large and slight changes in categories of ethical considerations. Claims made by different articles that constitute similar arguments were unified into single statements when we thought that this was fair with respect to the content of the claims that were made. Within single paragraphs, we have tried as much as possible (1) to group multiple statements that view an idea from the same perspective and (2) to contrast statements that provide different perspectives on the same point. Ethical aspects mentioned by papers were sometimes only asserted, yet sometimes supported by lengthy arguments. In order not to provide too much attention to long arguments, yet also not too little attention to short assertions, we applied two norms to the way in which we present ethical aspects. First of all, when an ethical aspect is hard to understand in (highly) summarized form, we have included (part of) the paper’s argumentation for the sake of clarity. Second, to indicate whether there is more (and which type of) content to be found (or not) in a specific paper on a specific ethical aspect, we have used words such as “claims”, “reports”, “argues”, and “discusses”. In order not to provide an unnecessary layer of interpretation, our descriptions of various ethical aspects often remain close to what is written in the papers that we cite. When authors have made claims in the context of a specific field or technology, we have specified that field or technology, such as metabolomics or epigenetics, when we think that this is relevant. In each case, we think that the claim is relevant to exposome research and could be extended to exposome research. Many ethical aspects discussed by authors (systematically) touch upon multiple issues, themes, and categories. To facilitate systematic thinking about the ethical aspects of exposome research, we have repeated a number of ethical aspects in multiple sections when necessary and added sentences in round brackets that cross-refer to other sections of the review when ethical aspects are interrelated.

## Theme #1: Research program goals

Exposome research is aimed at identifying what the exposome is, ie, what the various environmental (often characterized as non-genetic) factors are that have a biological effect on the human body (and what this effect is). As the exposome program is fostered within fields such as epidemiology and toxicology, this identification is intended to serve the underlying aim of improving human health (as opposed to, eg, understanding a person’s exposures to determine where they have been for forensic purposes). This means that the types of biological effects that are included in the exposome are selected based on particular health outcomes. In this respect, the goal of the exposome research program is to improve human health through the exposome approach. From this wide perspective on exposome research, various ethical considerations that are relevant to the whole exposome research program arise. We divide these considerations into a “research program” level and a “research project” level. The research program level concerns the way in which the goals that the exposome research program wishes to achieve align with other goals that are deemed valuable (such as policy goals or values from the funder). The research project level concerns the question which general considerations should be incorporated when particular exposome projects are being set-up with concrete goals, such as the investigation of a particular health outcome, time slice, or community.

### How do research program goals align with other goals?

Several papers mention that there is a need to assess the relationship between the goals of a research program (such as exposome research) and further (moral) goals or values. How do these align? Four papers provide relevant considerations. First, omics science has the potential to contribute to human well-being through economic, health, and social development.^9^ A second paper argues that biomedical research should develop a robust system that ensures the full societal benefits of research while respecting both individual needs and the communal good.^10^ The third paper argues that we should uphold the principle that the biomedical enterprise should aim at alleviating human suffering, mitigating environmental harms and generally improving the human condition.^11^ The fourth paper stresses the importance of the need to univocally assess disparate research fields with different goals that are united under a single program from an ethical point of view.^12^

### What general considerations should we incorporate into the goals of research projects?

Various authors outline considerations that relate to the ways in which particular research projects should incorporate certain values or goals. The first paper argues that ethical theory should help researchers to select the least harmful projects that most improve health on a global scale.^13^ Another paper poses the question whether there is a need for biomonitoring projects to be designed with specific policy goals in mind.^14^ On a more critical note, one paper argues that researchers need to be aware of the way in which research goals are affected by societal, market, health and environmental, and policy and regulatory demands.^15^ A fourth paper focuses on the pressure that can arise from the fact that omics technologies are developed within a laboratory context that epidemiologists are not familiar with, which may cause the omics technologies themselves to drive research instead of these technologies being a tool for well-trained epidemiologists.^16^ The fifth paper argues that medical research uses concepts such as “person” in different ways that might conflict across projects. Thus, they pose that research goals should consider the epistemological trends of medicine in order to have the clarity required for assessing research goals.^17^ Lastly, one paper argues that if scientific research is publicly funded, it should question its assumptions, impact on communities and individuals, and include communities, individuals and other “stakeholder” voices in the scientific process.^11^

## Theme #2: Research standards

In general, scientific research requires the use of standards to ensure the quality of research. Because exposome research aims to unite concepts, methodologies, and technologies from other lines of research and invent new research tools, it might be the case that existing standards do not fit well, or require an update. This theme brings together ethical considerations that directly relate to quality checks on research, whether they concern assay validity, peer review, ethics committees, or training programs for aspiring researchers.

### Measurement technologies

With respect to standards for measurement technologies, one paper argues that to prevent the neglect of existing knowledge, erroneous interpretations of studies, and false positives, we need to make sure that we develop standardized controls for high-content/high-throughput technologies.^18^ On the other hand, another paper points out that we need to make sure that standards and protocols still permit the dynamic development that is required for innovation in the field of omics research.^9^ Relatedly, one paper argues that the fast development of assays and tests can cause excessive consumerism and risk the inappropriate use of laboratory testing. Therefore, the paper argues that laboratory professionals that are familiar with both assays and tests should always be involved in research.^19^ In the context of clinical proteomics, one paper reports that, although standards and quality control may not intuitively evoke ethical questions, many articles on proteomics view meeting appropriate standards as a cornerstone of ethical proteomics because the clinical utility of proteomics is dependent upon the quality of the underlying science. The paper argues that to see that such standards are an ethical issue, consider that participants, patients, and the larger research community implicitly bestow trust on a published research project, which can be violated by incorrect, substandard, or nonvalidated methods, when better alternatives were available.^20^

### Peer review and editorial policies of journals

When it comes to the publishing of research articles in journals, several papers note that it is not feasible for journals to recruit reviewers with the expertise required for peer review to cover all of the details of multidisciplinary omics techniques that are paired with extensive bioinformatics.^18,21,22^ In the context of omics-based diagnostic and predictive tests, another paper argues that, due to the pressure to publish manuscripts in the most prestigious journals with high impact factors and editors who point out that the responsibility for articles rests with the authors instead of unpaid reviewers or editorial staff (especially for articles with complex computational aspects or big datasets), we need a register for the data, metadata, analysis plans, code, and fully specified computational procedures in a standard format.^23^ Similarly, in the context of bioinformatics, another paper argues that we need to simplify and automate the creation and storage of files, and details of statistical analysis from study data to ethically safeguard the computational reproducibility of statistical analyses.^24^ One article argues that there is no good way to let the scientific community know if there is a problem in a published paper because it requires a large amount of work to convince a journal to publish comments that criticize published papers.^21^ In response to these types of problems for omics science, two articles propose a post-publication peer-review process as an idea that might help to solve some of them.^18,21^ One article warns editors of journals to be aware of the violation of the ethics of the review process that is posed by researchers in proteomics who subdivide the advances of their projects into separate papers that present incremental advances and lack proper and complete referencing.^25^ The last paper issues the general warning that real-time online publishing of (omics) results can endanger the control that peer review has over the scientific process.^26^

With respect to the issue of data fabrication, one paper suggests that publishers should consider mandating the submission of all data to fraud monitoring to facilitate the detection of fabrication in large-scale molecular omics data. The paper also argues that journals should have a higher standard for data accessibility than a statement to the effect that “data will be made available upon request to the authors”.^27^

Two papers address the responsibilities of researchers when submitting articles to journals. The first article argues that, because reviewers are already overworked and metabolomics-based research (an omic-technology) is increasingly used as a justification for large clinical or environmental interventions, it is important that researchers make sure that they address ethical concerns in their manuscripts before they send them to journals.^28^ The second paper claims that researchers in molecular epidemiology and other biomarker-based research have an ethical duty to report findings with accuracy, completeness, transparency, and in sufficient detail to allow the scientific community to consider them adequately, assess their strengths and weaknesses, and make fair comparisons. The authors present an extension of an existing guideline for reporting in observational research.^29^

### Ethics committees and institutional review boards

Many institutions have ethics committees or institutional review boards (IRBs) to safeguard the ethical aspects of research and/or to provide other types of quality control for research. On this topic, one paper notes that ethics committees need to make sure that they have the specific competencies required for assessing biomarker studies and that these required competencies can vary widely per marker.^30^ Another paper engages in a structural critique of the way in which the ethics review system currently operates. It notes that, because the current ethics review system unduly and globally impedes advances in human health, we need to address its problems (such as presumed participant vulnerabilities, the lack of institutional support, and their ossification and overpoliticization).^31^ In reference to eHealth, one paper mentions that research tools and kits such as Apple’s ResearchKit, which allow researchers in the USA to conduct medical studies on iPhones, can provide data points related to the human body, behaviors, and their correlations for an extended period of time. The paper argues that such technologies hold incredible potential for science, also because they seemingly bypass extensive proposal writing and ethics committee assessment.^26^ Relatedly, one paper argues that due to regulatory gaps, researchers, and IRBs have little guidance on how to manage consent, expectations for privacy, and strategies to reduce risks of a data breach, when using eHealth tools such as pervasive sensors and/or social media platforms. It goes on to argue that, when using commercial products of which the quality is unknown, potential risks are introduced such as wrong usage by the participant or whether the data are owned by commercial entities. The paper argues that such complexities might not be well understood by IRB members if they lack expertise and that this could lead them to accept a proposal without a careful understanding of risk or reject a new and potentially fruitful line of research by applying standards from noncomparable research or fear of the “unknown unknowns”, whereby they squelch innovation. Lastly, it argues that, because ever-changing technologies create potential harms that were not present or known when a project first starts, we need to involve social and behavioral scientists in the development of responsive ethical guidelines and make use of platforms such as the Connected and Open Research Ethics (CORE) initiative for conversations and guidelines about digital health ethics.^32^ From a broad perspective, one paper argues that, in cases of scientific uncertainty caused by lack of descriptive accuracy and lack of action-guiding principles of traditional approaches, protocols, and paradigms, we should give IRBs and ethical theory a role to play in guiding researchers.^14^ Another paper reports on challenges for obtaining IRB coverage for a community-based participatory research environmental justice project. They found that IRBs sometimes unintentionally violate the principles of beneficence and justice, and conclude that IRBs and funders should develop clear and routine review guidelines for these types of projects.^33^

### Institutional policies and educational standards

Next to ethics committees and IRBs, research institutions also have policies when it comes to providing a good workplace environment and quality education. On this topic, one paper argues that research institutions should provide adequate protection, and research integrity standards that are suited for quality research, in particular for the protection of vulnerable biostatisticians and bioinformaticians from powerful principal investigators that might push them to find methods that support the principal investigators’ desired findings.^23^ Lastly, three papers argue that the development of new tools in biomedical informatics should prompt professionals to reevaluate their training programs for future researchers and practitioners and specify the competencies that are required for experts in the discipline.^34–36^

## Theme #3: Research tools

Exposome research aims to import, improve, and create tools that allow for better data gathering, storage, and analysis (such as smart sensors, databases, and algorithms). In this theme, we have gathered the ethical aspects that are relevant to the various tools that are being used, imported, improved, and created by exposome researchers. These tools are valuable for doing exposome research and are not ethical principles in themselves (such as privacy or justice). However, because these tools are one of the main vehicles of exposome research, their value relates to many of the ethical aspects of exposome research. Therefore, these tools are often at the causal nexus of different ethical aspects, which makes it important to thematize these tools in their own right.

### Expotypes and agnostic screening

The exposome research program advocates the usage of agnostic approaches for discovering and tracking exposures and their effects on the human body, as opposed to analyzing environmental health by looking only at specific exposures and their biological perturbations. The agnostic approach utilizes high-throughput/resolution methods and big-data analysis tools to identify whole groups of exposures and their correlations to each other and health outcomes. Exposures that are part of the exposome and are clustered together are aptly named an “expotype”, analogous to haplotype, which is a physical grouping of genomic variants (or polymorphisms) that tend to be inherited together. Expotypes are usually determined on the basis of large datasets and are an important part of the toolkit of exposome researchers.^37^

In the context of data minimalization, one paper argues that researchers should only gather the data necessary to answer a particular research question, while also including a specification and justification of the purpose for collecting data, to protect study participants from the effects of data leaks.^26^ Such a principle of data minimalization can conflict with big-data- and nonhypothesis-driven approaches that are appropriate for exposome research. Within the context of plasma proteomics, one paper notes that, although stripping data to such an extent that only disease-relevant information is left over might be necessary in certain diagnostic settings, it negates the possibility to derive maximum information from data to improve research, disease diagnosis and general health and well-being.^38^ Another paper notes that analyzing groups of exposures has particular value in more effectively regulating toxic chemicals.^39^

### The value of data sharing and integration

Exposome research aims to analyze all of the exposures that someone encounters throughout their lifespan, including the biological responses that their body has to those exposures. This is why exposome research requires data from a multitude of sources outside and inside the body, such as food, air, and various omics. To arrive at a comprehensive analysis of the exposome, researchers need to recognize not just the direct natural-scientific importance of sharing and integrating data, but also the broader value of sharing and integrating data for the scientific enterprise. Three papers take a wide perspective on this issue. The first paper argues that because the success of precision medicine depends on the availability of healthcare and biomedical data, it is essential that patients agree to share their personal and health data.^40^ The second paper argues that the scientific transformation in the era of high-throughput omics technologies is partly attributed to research data practices across studies, institutes, and international borders.^41^ The third paper argues that open data/sample sharing is necessary for scientific development, facilitates the harmonization of international database consortia infrastructures, and facilitates the achievement of scientific community goals such as replicating results, promoting new research, improving methods of data collection and measurement, enabling the teaching of new researchers, and allowing for more effective use of researchers’ and funding agencies’ limited financial resources.^42^

Data sharing and integration can also be valuable for achieving values other than (direct) scientific success or transformation. One paper says that benefits of data sharing are verification, data replication, the ability to pool analyses, and potential cost savings.^43^ Another paper says that data sharing allows the scientific community to be transparent and the scientific process to be reproducible and accountable.^41^ Similarly, one paper argues that benefits of data sharing are that it advances health science, reduces waste, allows for the validation and replication of research results, promotes scientific rigor, transparency, and accountability in science.^44^ One paper summarizes a number of ethical guidelines for the responsible collection and analysis of precision health data and presents consent policies, ethics guidelines, and privacy policies for computation on distributed precision health data.^45^

A number of papers comment on the value of integrating data from different sources and across exposure sources and biological strata (such as omics). One paper notes that the data mining of electronic medical records has the potential to establish new patient-stratification principles for revealing unknown disease correlations. However, it states that a systematic analysis of such records is blocked by a broad range of ethical, legal, and technical reasons.^46^ Another paper argues that, because most health conditions have low prevalence and an adequate number of records are needed to attain statistically relevant results, it is important to integrate records from multiple sources.^47^ One paper warns of the dangers of increased disease stratification by arguing that focusing on ever smaller groups with disease subtypes that were formerly indistinguishable by clinical methods, but that can now be precisely defined by biologic measurements, may impede the study of broad, general patterns, and mechanisms that different forms of diseases have in common, and limit appreciation for widely applicable and overarching principles of causation or treatment.^48^ Relatedly, one paper notes that 21st-century omics sciences and technology highlight the necessity of pooled data to engender value and a knowledge commons in the bio-economy, help investigate both rare diseases and common complex diseases with greater confidence, and provide much-needed statistical robustness and greater granularity.^42^ Two papers relate data integration to specific ethical values. The first paper mentions that a consideration for using pooled data samples in human biomonitoring and exposomics studies is that doing so may not require asking participants for consent and communicating study results to them, if, eg, individual samples are de-identified before pooling.^49^ The second paper argues that, to help address responsible innovation for a fairer and more transparent society, we need to generate metadata that shed light on how omics research is carried out, by whom and under what circumstances. It argues that doing so connects data to their production context and will create an “intervention space” for the integration of science within its sociotechnical context. The paper continues by claiming that metadata should not just be gathered on technical data, but also on theory-driven knowledge domains related to robust science, such as normative philosophical and bioethical analyses of emerging technologies and their policy recommendations.^50^

Several papers suggest or argue for ways in which responsible data sharing and integration could be realized. One paper says that, to maximize the (clinical) value of biomedical (meta)data from high-resolution medical imaging, behavior, wearable instruments and smartphones, and symptom/phenotypic data derived from social media, we need data to be digitized, integrated, structured, centralized, secured, and standardized (DISCSS), for which we need dedicated, integrated, and large-scale biomedical data management platforms such as TranSMART, FAIRDOM, and others.^51^ Another paper argues that an ethically robust way to share and harmonize data is by using the DataSHIELD approach, because it enables researchers to analyze individual-level data from multiple studies or sources without providing direct access to any individual-level data. This approach would contribute to protecting privacy, confidentiality and rights, and help address post-data sharing concerns.^52^ Relatedly, one paper presents a governance infrastructure that tries to incorporate independence, transparency, interdisciplinarity, and participant-centric decision making for the responsible sharing of (omic) data.^43^ To facilitate data sharing, one paper argues, we need sample procedure standardization and harmonization because this increases the effective sample size and statistical power (especially for rare diseases).^53^ (See also the section “Measurement technologies”.) Another paper argues that to merge data that has been generated in different environments, deep involvement of relevant stakeholders along the data-generating process chain is needed to better understand, manage, and mitigate quality (and data privacy) risks.^54^ One paper comments on cloud computing: because cloud computing allows for outsourcing and offshoring data practices, there can be difficulties relating to the control of the flow of data. The paper argues that this presents certain challenges, such as data control, data corruption, infrastructure failure, unavailability of data when required, questions concerning liability, and questions concerning the legal status of data across jurisdictions. Therefore, the paper argues, organizations and researchers should complete due diligence checks and negotiate with a cloud computing service before contracting with them.^55^ Another paper argues that crowdsourced research and development presents challenges for ethics and quality issues, such as potential bias,data quality, and scientific validity. The paper claims that these issues require effective mechanisms of ethical oversight, as can be seen from the uBiome project (a citizen science crowdfunded project mapping the microbiome).^56^ One paper argues that, for the clinical translation of multiomic data into personalized treatment strategies and risk management, AI serves as the central technology of a triad formed by patient data management, healthcare application, and services. It claims that accessing worldwide datasets facilitates recognizing and diagnosing rare diseases, which otherwise would have possibly never been identified.^57^ Another paper discusses techniques for the protection of the privacy of encrypted data that needs to be decrypted during computing tasks.^45^

One paper asks a number of general questions about data-sharing practices. First, when should researchers share data produced by array-based and high-throughput technologies? Some argue for early release of data, others argue for release by the time the data are published in a formal manuscript and a third group worries that releasing data ahead of publication leaves them vulnerable to being scooped. Second, it points to the question how long data should be shared. It notes that researchers who rely on controlled-access datasets often complain about periodical renewals for access.^58^ Another paper reports survey findings of women who volunteered to be contacted about breast cancer research for sharing different types of exposome/environmental health data. The authors claim that these results highlight three ethical imperatives for environmental health studies and exposome efforts. First, to respect and support participant motives and their desires to receive personal results. Second, to prioritize secure data access for researchers and have clear communication with participants on the data security measures that are being taken for their data for cases in which there is data misuse or a data breach. Third, if data are shared, to take steps to protect privacy and discuss re-identification risks with potential participants.^59^

### Data sharing “vs” other values

Next to the value of data sharing, many papers argue that there is a potentially problematic connection between data sharing and other values (such as privacy, participant integrity, or data security). This potential conflict is framed in various ways, such as the need to balance issues, to weigh different interests, or solve (apparent) conflicts.^10,20,40,41,44,60–68^ One paper argues that sharing genomic and some other “omic”-type data produced by high-throughput methods accelerates research progress, has been transformative for the scientific enterprise and benefits the public. Still, the paper argues, since there is an ethical responsibility to ensure that such data are maximally utilized for research (as public research funding needs to simulate the greatest public good), such data sharing must appropriately protect participant interests (such as privacy). Also, to balance most effectively the benefits of broad data sharing and the imperative to respect and protect research participants, it argues that there is a need to have a dialog within the research community that involves the full range of stakeholders.^10^ Similarly, another paper argues that, although the open science model has helped progress omics research, the private sector is concerned about intellectual property rights, data producers are concerned about attribution and recognition of their work and privacy advocates are concerned about privacy issues and data misuse. To address such concerns, the authors argue that we need to evaluate “controlled access” approaches that are part of an overall data privacy protection framework based on a tradeoff analysis.^69^ In that vein, one paper argues that fully open proteomics and metabolomics data sharing are incompatible with protecting identifiable patient information, and that we need controlled data access models for clinical proteomics and metabolomics.^70^ On a more critical note, one paper discusses the value of controlled access to proteomics data for the protection of privacy and makes recommendations on how to protect privacy in the future. It warns that making most human-sensitive proteomics datasetscontrolled access would undermine the research community’s culture of open data sharing and consequently hinder progress in proteomics and its biomedical applications. The paper also warns that a demonstrated high profile breach of privacy could lead to a severe backlash in data-sharing policy and erode public trust in proteomics research.^71^ (For a (potential) example of such a “high profile breach”, see the last part of the section “Forensic science and exposome research”.) Another paper points out that, although international research funders encourage sharing data to maximize discovery and innovation in public health, scientists are reluctant to share data due to various issues concerning topics such as intellectual property rights, data misuse and misinterpretation, privacy, confidentiality safeguards for scientists, unfamiliarity with data management systems and metadata standards, and a general lack of scientific culture for data sharing.^72^

### Study design and evidence

#### The principle of equipoise

Two papers discuss the relationship between the ethics of study design and the evidence that is required to justify scientific knowledge, which is typically at issue in discussions on the principle of clinical equipoise. The first paper argues that, aside from widely discussed issues such as informed consent, another ethical aspect is evolving around the issue of what kind of evidence is demanded for environmental health measures. In this context, the paper discusses the “lead paint abatement study”, an intervention trial designed to study whether less expensive abatement methods had the same effects as to reducing elevated blood lead levels. The paper reports that, whereas public health researchers argued that the proof for the effect of a cheaper abatement method could benefit large numbers of disadvantaged children in the future and that a randomized trial provides a high standard of proof, others argued that children were intentionally put at risk, and that research aimed at saving money, as well as such cost–benefit reasoning, is problematic in itself.^73^ The second paper argues that ethics and efficiency need to be balanced because they may conflict: Ethics may require us to minimize the number of subjects treated in the inferior treatment, whereas efficiency requires us to maximize the power of relevant tests.^74^

#### Bias in data, analysis, algorithms, and artificial intelligence

Broadly speaking, exposome researchers use data about exposures and biomarkers to reach conclusions about how those exposures affect the health of a person or population. If such a conclusion deviates from the truth, then there is something wrong with either the data or the analysis (or both). In this context, concepts such as exposure validity, biomarker validity, sensitivity, specificity, confounding, overfitting, underfitting, and statistical bias play a key role. It is important to distinguish statistical bias from “normative” bias because they are two different phenomena. Statistical bias is a cognitive notion that refers to the systematic deviation between statistical results and the truth, due to problems with data and/or its analysis (such as an algorithm, AI, or predictive modeling). “Normative” bias is usually understood as a form of prejudice or unfair inclination for or against a person or group. The two notions are related, as a statistical bias such as selection bias can lead to a “normative” bias when trying to assess a person or group in disregard of the statistical bias. To clearly distinguish between the ethical aspects of both senses of the term bias, we will primarily discuss the ethical aspects of statistical bias in this section, whereas “normative” bias will be primarily discussed in theme #5. Because papers often do not define the term bias, we have attempted to discern whether authors use bias in its statistical or “normative” sense and included its claim/argument on bias in the relevant section.

Many papers make the general point that having correct knowledge is a prerequisite for correct classification, and that a wrong classification leads to various problems downstream: the misclassification of exposure, biomarker or health status, or model overfitting or unreasoned/irrelevant algorithm selectivity, can be a major source of bias and can lead to incorrect conclusions about the association between exposure/biomarker and disease.^54,75–78^ In particular, one paper argues that if data classes are not represented equally, this imbalance fosters erroneous or reduced algorithm predictive performance, creating a bias in favor of data classes with a greater number of instances. It argues that features of minority class data may be treated by an algorithm as noise and ignored, or misclassified, causing the undervaluing of noisy or sparse data. It continues to argue that this can result in the algorithm missing important insights such as rare drug side-effects.^78^

A number of papers comment on the ethical aspects of selection bias. (See also the section on “Study participation” below, as these ethical aspects affect selection bias.) One paper argues that invasiveness, inconvenience, or physical discomfort of study participants during biospecimen collection may result in selection or nonresponse biases, which could affect the external validity of study results. It says that this type of error tends to distort the measured association between exposure and outcome so that the effect estimate is different for the subjects participating in the study from the estimate obtainable from the entire population targeted for study.^75^ Another paper claims that systematic underrepresentation of socially disadvantaged groups in environmental exposure studies undermines the external validity of scientific data and subgroup-specific analysis due to selection bias. The paper mentions that the most encountered participation barriers for socially disadvantaged people are feelings, resources, habits, and obstacles.^79^ Relatedly, one paper argues that differences in groups of people represented in clinical and quantified-self-data sources could result in a limited understanding of the range of symptom experiences and a lack of cultural context. As an example, it claims that because racial and ethnic minorities are less likely to participate in biobanks, discoveries based on omic data sources that may be of particular relevance to race or ethnicity are hindered.^76^ Also relatedly, one paper claims that omics research with underrepresented groups presents unique challenges based on historical ethical violations, such as the Tuskegee Study and the treatment of Henrietta Lacks.^80^ Another paper argues that research on gene–environment interactions has an interest to integrate different ethnic groups, as these represent different dietary habits and possibly different exposures. It claims that different ethnicities or religious backgrounds might stand for considerable differences in the extent to which the mother makes her own decisions or otherwise the father decides for her to participate in a cohort study.^30^

Several papers comment on the ethical aspects of selection bias from the perspective of “distributive justice”—which is an idea that we address more generally in theme #5. Three papers argue that recruitment strategies and study designs should pay attention to including certain groups to help secure certain aspects of distributive justice. They argue that diverse groups, underrepresented populations, low income, and various racial and ethnic communities should be included in order to avoid reinforcing health disparities, better understand and improve upon population health inequalities, and help address environmental justice.^78,81,82^ One paper warns of the ability of data, AI, and algorithms, as such, to reinforce existing socio-cultural discriminations that promote inequalities.^77^ Relatedly, another paper claims that the uneven introduction of AI technologies in the developed world and the systemic unavailability of AI benefits to the underdeveloped world are inherently discriminatory.^78^ One paper argues that socioeconomic participation bias in human biomonitoring studies is itself a form of environmental injustice because those who are most exposed and vulnerable are the least monitored and represented in research.^79^ Another paper argues that an equitable selection of subjects is an aspect of justice, because when a group is underrepresented in research, that group is unlikely to benefit from the knowledge that is discovered. It claims that justice encompasses recognizing that inequality in health status may reflect societal variables, rather than an exclusive focus on biologic variables (in research designs). The authors argue that we need to consider that meaningful variables in databases need to reflect potential sources of inequity such as environmental and sociocultural factors.^65^

Two papers comment on a potential feedback loop between selection bias and knowledge generation. The first paper argues that, because innovators and early adopters of big-data precision medicine are generally from higher resourced environments, this leads to data and findings biased toward those environments. It claims that such data generate new discoveries that obscure potentially underrepresented populations and create a nearly inescapable cycle of health inequity. The paper proceeds to argue for the idea that equitable access of representative data is of special moral importance to break the cycle of health inequalities.^83^ The second paper argues that existing health disparities contribute to unrepresentative training data, which may seep into predictive models and further exacerbate disparities due to biased predictions for certain minorities and vulnerable segments of patient populations. The authors claim that this creates a harmful feedback loop.^84^

One paper argues that researchers need to look out for the observational bias that occurs when researchers analyze data that are convenient to analyze, such as data that are already available to them (streetlight effect).^83^

Lastly, one paper tries to differentiate between desirable and undesirable biases. It argues that desirable bias implies considering sex and gender differences to make a precise diagnosis and recommend a tailored and more effective treatment for each individual. The paper proceeds to describe undesirable bias as exhibiting unintended or unnecessary sex and gender discrimination. The authors also present a list of six undesirable biases: historical, representation, measurement, aggregation, evaluation, and algorithmic bias.^77^ This article conflates the distinction that we have drawn between statistical and “normative” bias. From our perspective, using the (common) definition of statistical bias that we gave, we can say that the paper describes how statistical biases can lead to “undesirable” biases and how compensating for statistical biases can lead to “desirable” biases.

### Reference exposome

Exposome researchers aim to gather enough exposure and multiomics data to create a reference exposome.^85^ This tool will allow for network analyses across regions, population demographics, and other properties. It could also allow individuals to compare their exposome data to the reference exposome. Although the term “reference exposome” has not been used a lot in the literature yet, such a tool is currently being constructed by exposome researchers. In this section, we have gathered ethical aspects that are relevant to a future reference exposome. (See also the section “Public health and reference values”.)

Two papers discuss the value of population-level knowledge of exposures and associated biological responses using concepts related to the idea of “reference”. The first paper notes that we lack adequate information about “background” levels of exposure in the population, which are those levels that a statistician would call “normal”—the expected range of exposures in the general population. The paper argues that such background levels are important: it names a case where toddlers in a day care center were exposed to malathion, while there was only information about urinary malathion metabolites available from pesticide workers. Subsequently, the paper notes, there was no information on how to extrapolate from higher to lower exposures, nor information on how malathion metabolism might differ between toddlers and adults.^82^ The second paper argues that there is no such thing as “the typical individual” because every individual is unique (especially when disease is being defined at the molecular level). However, the paper notes, even though mean values are just abstractions, that does not preclude statistical analysis of grouped data. The paper states that, nevertheless, individual variability remains an important consideration for all statistical interpretations or risk management decisions where individual variability might be an issue.^86^

Several papers discuss the ability and value of individuals to compare their internal or external exposure information to group-level data. The first paper argues that participants may be able to learn about significant group risks if these are provided, but without risk functions that calculate individual risk, no meaningful individual information can be obtained.^87^ The second paper argues that comparisons of individual exposure results to a representative sample of their country’s population can lead to a normalization of problematic contaminant levels or cause people to mistake the exposure distribution of the population as a safety benchmark. The paper argues that this could give people a false sense of security or unnecessary concerns when their exposure levels are comparatively high.^88^ The third paper notes that clinical tests are obtained from individuals to make inferences about the etiology of disease at the individual level, whereas exposure biomarkers, although also obtained from individuals, are often used to make inferences about a risk at the population level. The paper argues that exposure scientists can effectively infer risk at the population level from biomonitoring data, but that such data are insufficient for determining individual risk. It goes on to argue that biomonitoring studies can yield evidence of exposures that often have no clinical importance, but are important to the public’s overall welfare. As an example, the paper says that low-level exposures to environmental lead are known to harm the neurological development of young children, but do not pose an acute medical emergency. However, the paper notes, the high prevalence of such low-level exposures does represent a public health threat, and small changes in exposures at the population level can result in large changes in morbidity and healthcare costs. Relatedly, the paper claims that an exposure biomarker is not clinically relevant until two empirical questions are addressed. The paper says that the first question is: What is the prevalence of this biomarker in the general population and subpopulations? The paper holds the second question to be: To what extent does the biomarker reliably predict susceptibility to, or presence of, a given disease? Subsequently, the paper also discusses the distinction between clinical and exposure science interpretations of dose due to conceptual differences and due to the fact that exposure science studies are usually observational in nature.^89^

In the context of epigenetics and the idea of a reference epigenome, one paper notes that researchers are characterizing the human reference epigenome. The paper mentions that these studies are expected to generate a list of detrimental epigenetic variants. Subsequently, the paper says, these studies determine what a “reference” or “normal” epigenome is: the epigenome that is associated with health or at least not associated with specific diseases. The paper mentions that defining epigenetic normality and abnormality has promising preventive and therapeutic opportunities, but is also scientifically and ethically challenging. Subsequently, the paper discusses a number of ways in which the ideas of “reference epigenome”, “epigenetic normality”, and “epigenetic ideals” might impact the construction of different types of personal and collective obligations.^90^ Relatedly, another paper reports on discussions in epigenetics on challenges when attempting to identify reference epigenomes and healthy epigenomes.^91^

### Intellectual property rights and patents

In the context of research tools, researchers relate to intellectual property rights in two ways. First of all, they sometimes have to make use of tools that other people have an intellectual property right over. Second, the tools that they develop might themselves be patentable. In this section, we have gathered the various comments on intellectual property rights and patents that we have found throughout the included literature.

In the context of omics-based predictors in clinical trials, two companion papers argue that intellectual property rights issues may be relevant to the use of specimens, omics assay platforms, in vitro diagnostic tests, and computer software used for calculation of the predictor. The papers claim that these intellectual property rights should be documented and respected by all parties involved and that potential conflicts of interest of study investigators must be disclosed and managed. Before developing a test, the papers argue, it is advisable to anticipate any intellectual property that may be generated in the development process and to agree in advance how it will be designated.^92,93^ Another paper mentions that metabolomics-based biomarker tests are patentable and argues that patenting specific metabolites for treatment purposes may be more challenging. The paper argues that this challenge arises because, for metabolites present in nature and already structurally described, only patents covering method of use or production processes would be possible. Still, the paper claims, chemical modification may be straightforward and lead to derived patentable novel chemical matter, which raises questions on who owns a trivially modified, but otherwise ubiquitous metabolite.^81^ Another paper argues that the use of human tissue/cells for research purposes raises questions regarding property rights and claims in terms of patenting. It claims that patients might express late and unexpected claims on products developed from their samples, which, in turn, makes researchers feel insecure when using samples of human origin and makes industry hesitate to invest in such research projects. The paper notes that academic research increasingly results in intellectual property protection and that academic patents are often licensed to profit-making companies. The paper proceeds to argue that the question to ask is not whether it is ethical to transfer human samples to profit-making companies, as the question is which person will receive a return on investment at which step of the value chain.^94^

Several papers discuss potential downsides or potential negative consequences that patents might have for other values. One paper argues that patents for biomarker tests are likely to impede progress towards integrating biomarkers into clinical practice.^95^ Similarly, another paper argues that patents are a potentially contributing factor to the problems involved in fully realizing concrete applications of omics research for human health.^42^ From a more positive perspective, one paper argues that it is essential to protect an established biomarker or panel of biomarkers by intellectual property protection and to provide the investors with exclusive rights over their work and discovery. However, the paper also claims that stringent intellectual property regulations often cause a major hindrance in trans-national sharing of scientific data, and that parallel research ventures on similar topics among multiple countries can be beneficial if these regulations can be liberalized to some extent.^67^ (See also the section “The value of data sharing and integration”.)

In the context of patent law in the United States, one paper mentions that new technologies cannot be patented if they are “obvious” changes to an existing patent. It argues that the definition of “obvious” thus has a huge impact on determining whether a patent is granted, such as modifications to microarray protocols in biotechnology.^96^ Another paper warns for so-called “patent trolls” in the field of proteomics and discusses how the United States Congress and Supreme Court are doing in their attempts to stop such actors.^97^

### General ethical aspects of biobanking

Biobanks are an important source of data in exposome research. Here, we will note some general ethical aspects of biobanking that are relevant to exposome research. This is not a comprehensive overview of all ethical aspects of biobanking, which is a topic that already has its own developed literature. Relevant values such as informed consent and privacy are discussed more generally in theme #4.

Three papers mention the importance of standards. To improve our understanding of human health across every “omic”, the first paper argues, we need to standardize the methodology of sample collection and storage.^98^ The second paper makes a similar point by stating that biobank policies need to be standardized, harmonized, and that governance structures need to be accepted by all stakeholders to ensure appropriate sample access for research.^53^ Similarly, one paper makes the point that biobank accessibility is challenged by inequitable access to high-quality specimens due to the complex level of control and ownership exerted by stockholders.^99^ The third paper argues that the standardization of operational workflows in biorepositories is a sine qua non for sound science and cannot be curtailed.^100^ Another paper makes a relevant counterpoint: that the regulation of the use of samples of human origin might hamper innovation in bio-medicine.^94^ One paper argues that, if environmental factors do not respect national boundaries and humanity is embedded in the total global environment, biobanks should be established on a national as well as an international basis.^101^

There are also a number of concerns with respect to the sustainability of biobanks. One paper notes that biobank regulations that protect privacy have been developed to protect the interests of the public, but do not keep in mind the purposes of the biobanks themselves, nor research interests and financial burdens.^99^ Another paper asks the question how biobanks can cover their costs if their commercial potential is constrained by ethical and legal issues.^53^ Relatedly, one paper questions how biobanks should determine when to throw away samples given the fact that it is hard to know whether they may become more valuable in the future due to new scientific developments.^94^ Lastly, two papers argue that we need to think ahead about what happens to samples, data, chains of informed consent for the continual use of samples, scientific consequences of losing rare sample types, and other problems when biobanks have to close down or otherwise need to eliminate stored samples and data.^98,102^

Two papers discuss aspects of biosafety when handling specimens. The first paper says that there are numerous biosafety concerns in terms of how a biospecimen is collected and the qualification of the working who harvests the biospecimen. The paper notes that universal safety guidelines for biosafety are hard to achieve or maintain in developing countries, as quality control is often compromised.^67^ The second paper says that biosafety aspects need to be addressed when researchers design studies using human tissues, particularly when international collaborations are intended or when collaboration between academia and industry is being sought after.^103^

## Theme #4: Study participants

Study participants in exposome research are an important source of the data that are required to study the exposome. For the purpose of this review, we use the term ‘participant’ as a wide category that encompasses any person that is an object of a study. They might be highly involved participants, patients in a clinic, people who only fill in a short questionnaire, or people whose registry data are being used. In this theme, we have grouped the ethical aspects that are relevant for proper research engagement with study participants.

### Distinction participant–patient and epidemiology–medicine

Currently, much of exposome research does not or does not yet have a clear clinical application, and data analysis tools that have to do with the validation of the effects of exposures on health and disease are still being developed. However, as exposome research becomes more clinically relevant, the distinction between research ethics and clinical ethics can be blurred and the duty of care for study participants can increase. In that context, one paper distinguishes the proposed response to elevated mercury levels observed in umbilical cord blood by the medical model and the exposure science model.^89^ Another paper argues that, because a human biomonitoring study is an exposure and uptake assessment study and not a clinical trial, a participant’s “right to service” has to be considered accordingly. In a clinical trial, the paper argues, the need for service encompasses treatment or use of a placebo. In the context of a monitoring program for involuntary environmental exposure, the paper argues, this need for service encompasses exposure mitigation for circumstances where high exposures are detected. The paper notes that the possibility of repeat sampling and/or interventions to reduce exposure has further impact on the participant’s “right not to know”.^104^ Touching on the participant–patient distinction, one paper claims that proteomic sampling procedures should not pose any risks for volunteers and only a low risk for patients.^103^ Relatedly, one paper notes that when it comes to community biomonitoring, no employer–employee relationship exists, and it is doubtful whether a doctor–patient relationship is present, as public health or environmental officials may be the driving force.^105^ Lastly, one paper argues that it is important that we need to take the distinction between clinical and “pure” epidemiological studies into account, as it is not a reasonable expectation that participants in these studies can distinguish the rights and obligations that follow from being a participant in a “pure” epidemiological study from the rights and obligations that follow from being a patient in a clinical study.^106^

### Study participation

#### Participant tasks and risks

What can researchers legitimately ask of participants when they participate in exposome research? One paper notes that, the more measurement instruments we apply to participants, the more that we burden them. The paper also notes that this might affect participation rates and cause a corresponding reporting bias.^107^ (See also the section “Bias in data, analysis, algorithms, and artificial intelligence”.) Another paper says that engagement with the perspectives of stakeholder communities within research and the involvement of stakeholder communities in decision making are lauded for meeting ethical expectations and norms, which improves the alignment of research with societal values and the relevance of research outputs or their translation.^43^

Three papers argue that we ought to pay special attention to the use of invasive methods. The first paper says that, because blood sampling is an invasive procedure, it is constrained by ethical considerations, especially when it comes to small children and other susceptible populations.^108^ The second paper adds that, because blood samples obtained from healthy babies and children have no apparent benefit to them, there is an ethical reservation with respect to obtaining parental consent.^109^ This argument relates to the question to what extent parents can rightfully have their child participate in research projects that both do not benefit their child and require invasive methods to be applied to them. The authors note that this issue can be avoided if it is possible for qualified persons to collect excess blood specimens from procedures that children do receive benefit from (assuming that parents provide consent for collection and usage).^109^ On a more critical note, the third paper argues that, due to feasibility and ethical reasons, human studies often require only minimally invasive biomarker collection, which is insufficient to capture the complex dynamics of epigenetic changes that may occur in specific tissues throughout development.^110^

Two papers comment on the potential consequences of data leaks for participants. The first paper warns for the possibility that data leaks cause knowledge of people’s medical conditions to become public, which can lead to societal discrimination, eg, by employers.^18^ In the context of proteomics, the second paper argues that one of the reasons privacy is important with regard to phenotypic information is the possibility that this information can be misused by third parties such as employers and insurers. For their purposes, the paper claims, phenotypic information is more interesting than genotypes alone.^41^

Lastly, in the context of Sweden, one paper mentions that insurance companies have the right to request medical information from, or authorization to access medical records of, a person who wants to take out life insurance. The paper mentions that participants who have had their omic data analyzed and are informed of pathological findings that require a follow-up in the healthcare system are documented in the hospital’s ordinary patient records. The paper notes that their participation thus might affect their ability to get life insurance.^111^

#### Participant rewards

Can we reward patients for participating in exposome research? Within the context of proteomics, one paper notes that when research requires economic valorization, reward mechanisms for participants can be legitimate as long as target participant numbers are reached.^41^ Another paper makes the general point that rewards for patients who provide tissue samples should be kept to a minimum.^103^ Building on the same point, another paper argues that providing financial rewards for patients that provide samples for research would open the door to the commercial handling of body parts and might encourage unethical sampling practices on normal volunteers.^94^

### Public trust in research

We found two papers that connect the issue of public trust in research to participants. The first paper argues that the willingness of individuals and communities to assume some risk to participate in biomedical research depends on the scientific community’s ability to maintain the public’s trust. It claims that patient-centric organizations and “citizen science” initiatives (such as PatientsLikeMe and Genomera) can promote participants’ long-term investment in and commitment to research, by which such initiatives gain public trust through transparency and accountability.^10^ The second paper claims that asking consent from participants is not just a means to protect researchers against legal claims but also a means to generate and maintain openness about research, and thus to enable trust in research.^41^

### Participant property rights: bodily materials and data

Individuals who choose to participate in exposome studies give researchers permission to use their bodily materials or information derived from their bodily materials for the purposes of scientific research. Legally speaking, there are laws that govern the gaining, keeping, usage, and disposal of material values, namely, laws that protect property rights. However, as one paper notes, ethical questions about whether a person has property rights in their tissues and its components and structural features are independent of and form part of the justification for national laws.^101^ Another paper notes that, although the International Agency for Research on Cancer states as a general rule that “no ownership of biological samples exist, and the biobank should assign ownership or custodianship based on national and institutional guidelines”, the question of who owns biological specimens remains an important unsettled topic discussed in the literature.^53^

To help categorize ethical aspects, it is important to have clarity about what the terms “bodily materials” and “data” refer to in this context. One author argues that we need to clarify and differentiate between multiple concepts of property such as real property (such as blood samples), intellectual property (such as gene patents), and informational property (such as genetic code) because otherwise we risk exploiting participants that are involved in big-data-centric science projects.^26^ Within the category of “real property”, however, some people believe that different disposition rights apply to different kinds of bodily materials such as urine, blood, organs, and other human tissues, because of, eg, the risk that is coupled with the extraction procedure. We must note that it is not always clear what types of bodily materials the various authors that we refer to would categorize under concepts such as “organic materials”, “tissue”, or “body part” because their usage of these concepts often lacks definitions and examples. Questions concerning property rights in bodily materials and data that are derived from one’s bodily materials are often intertwined in the literature. Below, we have tried to separate them as much as the logic and scope of the various comments allows for. Also, since concerns about the commercialization of bodily materials and data are mentioned as reasons to constrain the rights that people have to use bodily materials and data, we do not mention these in a separate category because we choose to focus on the moral rights of study participants here.

#### Bodily materials

One paper points out that the Council of Europe’s Convention on Human Rights and Biomedicine states that the human body and its parts (including blood) must not, as such, give rise to financial gain (thereby presenting a limit on property rights). However, the authors of this article argue, there is always value that is created through the highly complex value chain that connects a patient’s provision of the initial tissue and the final sale of a drug. Thus, they argue, the correct ethical question is not “should financial gain be allowed?” but “who will receive a return on investment at which step of the value chain?”.^94^ Similarly, one paper presents a case that shows a lack of transparency in the commercialization of tissue samples. The paper says that many European countries sign documents that affirm the noncommercialization of the human body and regulate the issue by allowing for various forms of buying and selling of human biological material. Thus, the paper notes, the issue of commodification of biological samples highlights (1) the limitation of public knowledge about the transfer of human tissue for commercial use and (2) that commercial aspects are very often not explained in the informed consent process.^112^ Relatedly, one paper argues that human microbiome samples should not be subject to due diligence obligations under European Union Regulation 511/2014 because states have no sovereign rights over human microbiome samples or over their citizens. Thus, the paper argues, the sole owners of these samples are the individuals from whom they were obtained.^113^ Against such an idea, another paper notes that, in contrast to human genomic or epigenetic information, microbiomics data are obtained by the analysis of the genomic composition of nonhuman cells and as such may not be conceptualized as belonging to individuals. However, the paper argues, microbiome samples used to gather microbiomics data will contain human DNA and the data may or may not reveal host genomic sequences depending on the test used.^114^ Another paper holds that individual human beings should have ownership over their organic materials and the data derived therefrom (such as one’s genome, epigenome, proteome, metabolome, and microbiome), and concludes that scientists can only either license the use of this information or ask for meaningful informed consent.^11^

When it comes to the clinical context, one paper questions whether physicians have the right to use bodily materials obtained from patients, or whether patients remain the owners of the parts separated from their body.^103^ One author argues that patients remain the owners of their bodily materials, but can transfer ownership to physicians or hospitals or waive ownership when abandoning their bodily material without any disposition.^115^ Two papers argue that, because Guthrie cards are valuable scientific resources for epigenetic studies with the potential to benefit society, and because they often have an absence of clear rules of ownership, we need well-defined property rights to govern these cards (and preservation of patient autonomy through informed consent).^116,117^ Another paper questions whether the veto right of local clinicians or principal investigators to control samples in biobanks from patients affected by rare diseases or innovative clinical trials implies some sort of ownership of biological material that is disreputable from an ethical point of view. As a potential solution for this issue and to allow equal access to the collection, a “shared ownership” model is put forward. In such a model, biobanks provide free services to constitute the collection for a specific research project, but half of the aliquots collected will be used by the biobank for additional research projects when necessary.^53^

Three papers make general points about rights to bodily materials. The first paper provides a short discussion of how the academic debate concerning the ownership of one’s body and its constituents affects changes in public policy.^98^ The second paper points out that, in whichever way policy verdicts might come down on the issue of tissue ownership, from the start of a biobank, it needs to establish exactness about ownership rights, or the extent to which donors are deprived from such rights.^102^ (See also the section “General ethical aspects of biobanking”.) The third paper asks a number of questions on the issue of whether and to what extent the originators of human tissue have property rights over and should exercise control over those tissues.^101^

#### Data

As mentioned above, sometimes authors view data as something that one can hold as property. One paper points out that a data-related ethical question arises if we want to communicate scientific findings: Who owns community- or individual-level data?^14^ Another paper notes that patients have a right to their own epigenetic data and should be able to request their data returned later if they would like to withdraw from research.^118^ One paper points out that, if genetic and epigenetic information that might identify a person are the individual’s property, then this may interfere with biomedical research and undercut the utility of insurance in limiting insurers’ knowledge of risk-relevant data.^117^ Another paper argues that, with respect to digital twins derived from a patient’s omics profile, there are ethical concerns with respect to the ownership, privacy, and storage of patient data, as well as the shift to computation-aided health care.^119^ Lastly, one paper argues that we need the idea of multiomic dignity, because this would allow participants to own their omic data and has the potential to repair trust and advance health equity for otherwise excluded populations.^120^

#### Retraction of bodily materials and data

Questions that are related to property rights in bodily materials and data concern whether a participant has the right to retract bodily material or data from a study and the extent to which it is possible for data to be retracted from studies (also described as withdrawal, deletion, or erasure). One paper says that patients have the right to request the destruction of both their sample and the related data at any point in time during the course of the study.^103^ Another paper writes more extensively on the subject. It notes that, although it is widely accepted that people should be able to withdraw their consent from biobank studies, there are discrepancies in the literature about what it means in practice to terminate participation. It points out that, if samples are anonymized, then they cannot be withdrawn. It also presents a complication: It refers to a number of papers that argue that even coded examples cannot be withdrawn, such as when (double) coded samples are sent to collaborators on the other side of the world. It also argues that scientists must be honest with participants about the extent to which their samples and data can be destroyed upon their request, as not to give false impressions.^18^ On a practical note, one paper argues that the right to withdraw data can be denied to participants in large-scale international sharing projects due to the impracticability of keeping track of data.^119^ With respect to cases in which participants have made use of this right, one paper reports that in response to the use of research data from the PKU Swedish biobank by the Swedish government to solve the murder of the Swedish Minister of Foreign Affairs in 2003, many Swedish citizens chose to withdraw from the biobank in question and asked it to destroy their samples and erase their data.^112^

### Participant privacy

Many different papers express concerns about the privacy of study participants. We found no single view or definition of privacy that was being used across these different papers. However, many papers commented on situations where research (potentially) protects or violates the privacy of study participants. One paper notes that the dissemination or revelation of results beyond the explicit purposes for which specimens were collected intrudes on the privacy of subjects.^87^ Another paper argues that the privacy of a person is preserved when their name or other identifying characteristics are protected and when the researcher does not collect more information beyond that which is needed to meet the aims of the research.^65^ Within the context of epigenetics, one paper argues that the privacy of epigenetic data donors will only be violated when two conditions are met: data are identifiable/re-identified or can be brought to bear on the individual, and the data are sensitive/reveal something about the individual.^44^ In another paper, it is argued that there are two ways of protecting privacy: either by making sure that direct and indirect identifiers are not and cannot be linked to individuals, ie, anonymity, or by making sure that potentially (re)identifiable datasets are not revealing any sensitive information about persons unless implicit or explicit informed consent has been given for the usage of such information.^122^ Another paper emphasizes the point that risk levels need to be considered as well, by arguing that there is a need to adapt the degree of privacy protection according to the risk level posed by epigenetic data.^91^ Another paper provides an overview of views about privacy protection within the literature on epigenetics.^45^ One paper notes that one way of safeguarding the right to privacy and the right to benefit from science is to ensure a robust and independent data access process, especially for the most complex and sensitive research resources.^43^ Within the context of health care, one paper provides a survey of regulations, ethical guidelines around the world and domain-specific and situation-specific needs for precision health data security and privacy.^45^

#### New risks and protection mechanisms for participant privacy

Many different papers note how new developments in research might pose new/novel risks for the protection of privacy. One paper points out that as modern medicine shifts from general therapy to patient-specific treatment, data obtained from patient history are needed to interpret molecular data, which gives rise to sensitive privacy protection problems.^94^ Similarly, another paper says that the real-time monitoring and diagnosing of patients at any place or time raises possibilities for questionable use and abuse of private information, and drives public debates about health/privacy tradeoffs.^123^ Another paper argues that the increase in density and availability of meaningful genetics and omics information leads to a perceivable public trend, which shows that people are being desensitized to privacy concerns instead of becoming fearful for losing their privacy due to the perceived explosion in information sharing online.^124^ One author says that, although patients can benefit from the early identification of epigenetic changes, a fear of the loss of privacy may prevent them from taking advantage of these tests at an early age.^125^ Charting the wide-ranging effects of metabolomics, one paper argues that because metabolomic profiles can also be established from surfaces such as one’s phone or kitchen, and because metabolomic profiles can be used to build behavioral profiles, the metabolome (intersecting with exposomics) can cause a threat for personal privacy.^81^ In a discussion of biobanks, one author argues that there are potential problems caused by having biobanks that contain large sample sets and big databases, in combination with the usage of high-throughput methodologies, particularly when these are being combined during extreme competition and haste to commercialize research achievements. The author says that this may endanger privacy through hackers, intentional loss of data, sample, and data collection made without informing individuals, movement of hospitals’ sample sets into biobanks, or accidental misplacement of discarding of data.^18^ Similarly, another paper argues that privacy assurances for individuals become more complex because data are shared more, biospecimens may be used for purposes that could never have been anticipated at collection, and because interest groups call for access to datasets that have powerful societal implications.^36^ In trying to cut across a variety of omic data, one paper provides a list of privacy-relevant omic data properties for a general framework that allows for privacy risk assessments in multiomics research and databases.^122^ Commenting on this list, one paper argues that the identification of privacy-relevant omic data properties is a normative enterprise from the outset, and provides suggestions on how to accomplish this task.^126^

Five papers comment on ability of technological developments to better protect privacy. The first paper asks the question: How should we take into account new technological developments that can protect privacy? Its author argues that, to keep big biomedical data secure and private, we need to look into security systems and de-identification algorithms used by banks in the financial sector to secure client privacy as well as de-centralized storage and in-house storage options.^46^ The second paper argues that DataSHIELD technology is especially suited for privacy protection because results returned to the analyst can be carefully created to be nondisclosive and match the policies of the data provider’s data governance rules.^127^ The third paper holds that blockchain technology could provide a secure private information ledger where data providers (individuals): are in control, own their information and can monitor access privileges, and are informed about who accessed their information.^41^ Relatedly, a fourth paper discusses cost analysis, ownership, data collection, authorization, security, and anonymity issues for block blockchain-based platforms that allow the sharing of omics data.^128^ A fifth paper reports on end users’ perspectives on use of blockchain solutions for private and secure individual omics health data management and sharing.^129^

#### Privacy protection “vs” other values

Just as we found papers that argue that we need to weigh the value of data sharing and other values such as participant integrity or data security, we discovered papers that argue that we need to weigh privacy and other values such as innovation. (See also the section on “Data sharing ‘vs’ other values”.) One paper argues that there is a conflict between patients’ data privacy and the need for medical information. The authors argue that, although patients have a right to data privacy, this clashes with the more basic need of patients to live and to prevent death and disability, to receive effective medical treatment for their condition when the costs for state-of-the-art treatment are exorbitant, or when the current level of medical science cannot deliver effective care even at the symptomatic level due to the limit of knowledge and insight into pathogenic mechanisms.^61^ Another paper argues that, because omics technologies-driven research spawns a broad range of potential privacy issues that might include conflicts between privacy and other values, we need an expert and review body that encompasses the various interest groups.^26^ One paper argues that there is a huge ongoing effort to find solutions that balance the needs of AI-driven health care and privacy.^121^ Relatedly, one paper argues that standard personal health information data firewalls designed to protect privacy data from improper access generally restrict or prevent most machine learning and AI technology applications. As a solution, it argues that data security and transactional privacy could be maintained if data are stored via blockchain because (1) it stores data in an encrypted distributed ledger of transactions generated by a swarm of cloud-connected edge computing machines and (2) because a blockchain’s components can directly learn from each other without having to share data on a common cloud computing platform.^78^ Similarly, another paper claims that we need solutions to protect privacy while permitting data sharing and usage and argues that blockchain could be one of those solutions.^129^ In connection to this point, the first paper mentioned in this paragraph also discusses the capacity of blockchain ledgers, federated databases, and encrypted computation to protect the privacy of patient data and argues that these solutions all drive up overhead and research costs, limit the number and diversity of scientists that can participate, and require many software change approvals by audit when approaches and methodologies require new software adaptations. The authors think that these methods have their role to play when no other access to data is possible and that encrypted and federated databases become more useful when principal aspects of research methodology are already established.^61^

#### Exposomic and genomic exceptionalism

During the 1990s, there were authors arguing that genetic information is an exceptional, special kind of information that spawns its own special ethical concerns and requires special data protections.^130^ This idea has been termed “genetic exceptionalism” by an opponent of the idea.^130^ How does this debate affect exposome research? The paper that started the discussion about exceptionalism within epigenetics argues that, although the debate about genetic exceptionalism remains ongoing when it was published, it is still difficult to discern any unique principles that are differently applicable to epigenomics than they are to genomics. Thus, the paper makes the (ironic) observation that in rejecting epigenetic exceptionalism, policymakers might find it necessary to amend laws previously enacted under the theory of genetic exceptionalism.^130^ Two other authors remain more on the fence: they argue that there are salient differences between the genome and the epigenome, such as the epigenome being subject to change depending on exposure and stage of development, which leads to the conclusion that we still need to think about the extent to which there is a need for “epigenetic exceptionalism” or not.^117,131^ Another author argues that epigenetic data should be given the same protections as genetic data because of the complexity of the information that is revealed by epigenetic testing.^125^ One paper aims to demonstrate that privacy-impacting data properties are often shared across broadly defined categories and (multiomic) data, which would lead to the conclusion that we need to avoid any kind of exceptionalism.^122^ In response to that paper, two papers comment on the role of “contextual” factors in identifying privacy-impacting omic data properties for avoiding exceptionalism.^126,132^ One of these papers also argues that, to avoid exceptionalism from the outset of one’s privacy analysis, one should first ask the general question what a data property can tell us about what a person’s life looks like when determining the privacy relevance of omic data properties.^126^

#### The anonymization of data

To protect the privacy of study participants, one often-used method is the anonymization of data. Commenting directly on the exposome, one author argues that exposome research needs a secure and reliable system that permanently and completely removes personal identifiers from data so that they can no longer be re-associated with an individual in any manner.^34^ Other papers are critical about the feasibility of such anonymization. Eight papers warn about re-identification risks generated by advances in high-throughput methods or (multi-) omics data, with special reference to continued identifiability issues within genetics.^10,41,58,62,131,133–135^ Four papers argue that anonymization is hard or impossible to accomplish because it may be possible to identify individuals via triangulation, by combining data from other databases or online sources.^18,44,51,121^ One paper provides a statistical analysis of how much private data are contained in aggregate GWAS results and argues that new methods need to be devised for privacy protection in an era of multiomics data.^64^ Three papers argue that because (plasma) proteomes can be reidentifiable and provide information about personally sensitive data, we need to review the privacy risks in proteomics and propose solutions.^38,41,71^ In the microbiome literature, one paper aims to demonstrate that microbiome-based identifiability is possible for a nontrivial fraction of individuals in a typical cohort, even though the relevant microbiome features are generally less unique and stable than features of the human genome.^136^ One paper provides a general comment on re-identification research and argues that ultimately, the adequate level of (omic) data (privacy) protection should be determined by considering the scientific, social, and policy context and following a thorough risk–benefit analysis of the research being undertaken. The authors argue that, ideally, re-identification research should consider not only the technical potential to achieve re-identification but also the full spectrum of administrative, legal and information technology measures available to reduce the existing risk.^137^ Another paper tries to soften the blow of the difficulties associated with anonymization by arguing that anonymization or identifiability are not bimodal, but exist on a continuum that spans the likelihood of the re-identification of a person.^44^

There also seems to be a need to reflect on the effect on anonymization of the lifespan-level temporal dimension of the exposome, as two papers argue that modern (precision) medicine needs to track patient data for a length of time that excludes true anonymization of samples.^41,94^

Taking a broad perspective, one paper stresses that, when analyzing biological samples, we need both adequate anonymization and identification procedures: the former to protect privacy, the latter to enable researchers to contact donors in case of accidental and significant findings for the donor’s health. However, they argue, because deleting identification information is insufficient to protect privacy, pseudonymization is often used as an alternative.^102^ Similarly, another paper argues that the (theoretical)loss of the possibility for anonymization need not be problematic due to the possibility of coding or double-coding of data. They point out that coding also has scientific usefulness and potential personal benefits to the donor.^44^ As another paper notes: the standard routine in hospital-based biobanks is to de-identify individual samples with codes that can only be translated back to individual identities by the primary holders of the study permissions granted for samples to be acquired, stored, analyzed, and reported.^138^

With respect to the communication of privacy risks that are connected to anonymization, one paper argues that research participants should be informed about data security measures and encryption options posed by cloud-based repositories.^55^ Relatedly, one paper argues that unified definitions of terms such as anonymization produced by standardization activities in the privacy enhancing technology field tend to be very formal and exact, resulting in complex and hard to understand constructions and wordings and so do not lend themselves to use in communicating with patients.^47^ Another paper argues that to better communicate the risk that is actually incurred by participants in omics data-sharing projects, it could be worth contrasting the risk incurred by participants accepting the open release of their genetic expression data to the risk incurred in everyday life by a regular Internet user.^137^ Lastly, one paper says that in addition to efforts to maintain data security, one element of informed consent that addresses confidentiality is that individuals would be told whether or not identifiers will be removed from their biospecimens, such that they can make their decision to consent or not while knowing this fact.^65^

#### Traceability of data and tissue

As mentioned above, one paper argues that we need both adequate anonymization and identification procedures: the former to protect privacy, the latter to enable researchers to contact donors in case of accidental and significant findings for the donor’s health.^102^ This latter concern relates to the issue of the traceability of data and tissue. One paper defines traceability as the existence of a guaranteed continuous chain of responsibility in relation to the derivation, storage, handling, and use of body materials and personal data. Its authors argue that traceability needs to be ensured when it comes to samples of human origin.^94^ Another paper says that the traceability of the donor of proteomic material should be ensured to optimize the security level for both the donor and the researcher.^103^ Lastly, one paper argues that the complete anonymization of samples may be problematic from an ethical point because certain health information that is relevant to the research participants themselves may be discovered.^41^ This argument can be taken to support a continued need for traceability and pseudonymization.

#### Participant confidentiality

One paper describes confidentiality as referring to not divulging data without consent.^65^ We think that, because exposome research does not yet have a direct focus on the patient–physician relationship, where confidentiality is of high relevance, we found that most authors do not draw clear boundaries between privacy and confidentiality (with one clear exception, which we discuss in the section “Exposomic and genomic responsibility”). For example, one paper argues that because of the greater use of electronic health records and data repositories, the expanded health information created by epigenetic research and applications will be more easily accessible and thus spawn new privacy and confidentiality questions about access to and secondary use of epigenetic information in various settings, such as insurance and employment.^130^ Similarly, one paper says that confidentiality when handling sensitive patient information is important because the breach of confidentiality could subject the bodily material donor to stigmatization, discrimination, and other forms of harassment.^139^ Another paper connects the issue of confidentiality to the anonymization of data by arguing that if researchers are no longer able to guarantee that de-identified data will remain anonymous, then they cannot commit to the principle of confidentiality.^44^ One paper makes the point that if broad statements are made during the consent process to the effect that confidentiality will always be adhered to, if not accompanied by any further explanation of what this really means, then they are not effective disclosures in terms of informed consent.^133^ Another paper states that researchers must consider in advance how the confidentiality of an individual’s results may be affected by the reporting back of results: if individuals learn their own results, then they might be obligated to disclose them to others.^88^ This last comment brings us to a related topic in the next paragraph.

#### Exposomic and genomic responsibility

One paper mentions that some ethicists have argued for a moral “genetic responsibility” to share medically relevant information with biological relatives who have an interest in this information, such as family members who share similar genetic risk profiles. The authors argue that such a moral responsibility can conflict with the obligation of physicians and researchers to protect patient confidentiality and that the superior approach is for healthcare providers to council, encourage and support patients to disclose relevant genetic information to their at-risk relatives. In their exploration of a notion of “epigenetic responsibility”, the authors point out that the effect of environmental exposures on epigenetic changes would require us to expand the notion of “biological relative” to include “individuals with shared exposures”. They take this category to encompass individuals who are likely to share similar epigenetic risk factors which, they argue, could potentially include family, people in one’s environment, as well as their future children if there were a risk of possible transgenerational effects.^140^ Relatedly, another paper mentions that, if a participant has been given individual risk information based on molecular epidemiological research, they might have to inform their life or health insurance, which could lead to discrimination in healthcare availability or in the workplace.^141^ If a notion of exposomic responsibility exists, then it would have to take similar (environmental) concerns into account and differentiate whether and to what extent such a responsibility would be held by individuals qua individual, study participant, patient, researcher, or doctor. (See also the section “Exposomic actionability for individuals” below.)

### Informed consent

Nearly all the papers that we found that mention the value of informed consent make statements about its importance in general terms or cite existing guidelines on informed consent. In our section on informed consent, we have grouped ethical aspects that go beyond such statements and are relevant to exposome research.

A number of authors comment on the context of informed consent or the intended limits of informed consent. One paper argues that community-based participatory research does not mean a hands-off ethic for researcher with respect to the principles of beneficence and nonmalfeasance, because informed consent offers an opportunity and responsibility for researchers and community partners to jointly articulate the potential community and individual benefits and harms considered by the study team as possible sequelae of reporting results.^88^ Another paper argues that although in the everyday proteomics research practice it is the case that regulatory aspects, access to adequate samples, privacy concerns, property, and value creation aspects are best addressed by requesting informed consent from a research subject, informed consent does not in itself ethically justify putting subjects at risk.^94^

#### Waivers of consent

Two papers mention waivers of consent. The first argues that, if omics research risks are minimal, which means that subjects are put at levels of risk no greater than those experienced in everyday life, then researchers may seek a waiver of consent from the appropriate IRB.^142^ The second points outs that waivers for informed consent to use medical data are uncommon and exceptional, but could be given by ethical review committees when obtaining consent is impossible or impractical.^47^

#### Making consent informed

A number of papers discuss the way in which it can be established that the consent provided by a study participant is informed. One paper states that ensuring that each subject understands the implications of participating in a study is difficult and there is no simple formula for developing consent forms.^87^ Another paper argues that informed consent challenges biobank research because relevant information is not known or not clearly stated at the time of eliciting consent.^112^ Two papers build on this observation by arguing that informed consent processes need to be revisited to be adapted to reflect current technological and scientific practices, such as when omic data confidentiality promises become unrealistic.^137,142^ Similarly, one paper argues that, because the language of omics research is complex, it is important to assure omics literacy and confirm understanding among potential research participants to protect the consent process.^65^ Another paper argues that terms of service of social network sites and other quantified-self technologies are usually lengthy, may not highlight use of data for research or commercial purposes and may not be fully comprehended by users. Consequently, the paper argues, individuals might technically consent by clicking “I agree”, but such consent does not meet typical research criteria. The paper thus argues that approaches for informed consent need to be reconceived for research in the social-computing environment.^76^ Commenting on what informed consent documents have to contain, two companion papers argue that informed consent documents for clinical trials using an omics test must accurately describe any potential risks from participation in a study, all potential conflicts of interest on the part of study investigators or sponsoring institutions and allow for “bridging studies” to validate new or improved assays.^92,93^ Another paper points out that informed consent in clinical and research contexts may be complicated if new medical nanotechnologies require individuals to make a risk assessment in the absence of adequate information about nanotechnology-related risks.^123^ A paper that also discusses risk found that information on the risk and the possibility of suffering from cardiopulmonary disease was perceived very differently by individual participants, which prompted researchers to change the informed consent form and revise the information given to them.^111^ A paper that also discusses the perception of study participants argues that, because some people are concerned about data sharing for commercial gain, research involving other medical conditions, sensitive research such as epigenetic analyses, or because (some) people have (personal) reasons to withhold consent such as when research is done in indigenous populations, it is not self-evident that, when people consent to secondary use, they also agree to these types of use.^44^ Another paper states that informed consent forms for prospective studies should specify various data usage options and include an expiration date after which samples and personal health information will be destroyed.^142^ One paper outlines six options that researchers could offer to study participants for consenting to the use of their proteomic materials for research, such as coded or identified use for a single study, or permitting usage for any future studies. The paper also argues that donors should be informed up front that they do not have any commercial rights on potential research results that lead to novel therapies or patents.^103^

#### Different types of consent: broad, dynamic, and open

Many papers discuss the need for and value of different types of consent. One paper argues that, because an a priori definition of future research projects to be performed with tissue from biobanks cannot be given, it is in the researchers’ and community’s interest to keep the definition of the field of research in informed consent as broad as possible in order to be able to research as widely and as intensely as possible.^94^ Another paper says that the nature of informed consent has changed in many countries: from participants acknowledging that they accept potential risks associated with giving a sample for a specific purpose at the time, to a broader consent that asks participants to agree to the use of their samples to be stored for an unspecified time and used for unspecified assays at some time in the future.^141^ Another paper argues that the need to obtain explicit patient consent for the use of routine clinical data can be resource intensive and lead to biases as a result of differences between consenters and nonconsenters. Thus, they argue, we need to examine whether and to what extent researchers can access patient data without consent.^143^ (See also the sections “Bias in data, analysis, algorithms, and artificial intelligence” and “Waivers of consent”.) One paper argues that omics data research challenges fully informed consent because secondary data processing or re-processing according to the FAIR (findable, accessible, interoperable, reusable) principles conflicts with data processing within the frame of duration-defined specific purposes.^51^ Another paper argues that asking consent for a single clinical trial instead of a broad consent that fully anonymizes data is a waste of economic resources as especially omics and image data that are collected might be useful for unrelated research.^61^ One paper connects the discussion on the acceptance of risk by an individual via informed consent to risks on the societal level. If providing informed consent for donating to a biobank implies accepting a risk/benefit analysis that affects society, then is it right to give such societal responsibilities to individuals?^112^

A number of papers specifically discuss informed consent for biobanks. Two papers report that there is no consensus for the type of informed consent that should be acquired for the biobanks that are used in omics research.^18,139^ Similarly, one paper points out that the answer to the question whether or not specimens collected for one purpose can be used for related or for distinctly different research has not been clearly delineated for retrospective studies.^87^ One paper argues that biobank donors should be provided written information such as type of samples to be donated, duration of storage, type of research, access to the samples, right to withdraw from participation, and possible intellectual property rights that may result from research results.^144^ Another paper presents a framework for integrating biobanks into national eHealth ecosystems that would facilitate the ability of citizens or patients to quickly review and take informed decisions about providing consent for specific research experiments.^145^ Two papers argue that classical informed consent is insufficient in biobanking due to the limitation of sample use for one specific project. To solve this problem, it is said that general/broad consent is formulated: a patient’s agreement for the utilization of their sample for current and future studies within a specified framework without the need for recontacting the patient.^53,102^ However, one paper says that obtaining informed consent for each project that a sample in a bio-repository may be subsequently associated with could be a logistical problem.^67^ Another paper argues that, because nobody can anticipate the type of information gathered from biobank samples nor predict who can access them, we need either a well-managed broad consent or update original consent forms over time.^142^ These two types of informed consent are called broad consent and dynamic consent, which will be the topic of the subsequent two paragraphs.

With respect to broad consent in particular, one paper argues that, because omics research includes the possibility that research samples may be used in unforeseen future studies, broad consent must be considered, which is intended to give permission for the usage of personal information or biospecimens that were originally obtained for purposes including original research and/or clinical care for reusage in the future for research that cannot be described.^65^ Another paper states that broad consent requires mechanisms that ensure that these consents and their expectations are maintained, such as an explicit statement of which bodies can approve data and sample access.^43^ One paper lists various practical considerations that researchers can consider when thinking about incorporating broad consent into their omics study.^65^

With respect to dynamic consent in particular, one paper argues that dynamic consent enhances autonomy and helps meet the desire for increased user participation in research programs.^53^ Two other papers argue that dynamic consent requires digital tools for easy accessible constant contact with the patient in order to manage re-consent for each new research.^41,102^ Another paper argues that, although dynamic consent can be helpful for reusing, sharing, and linking data, it also comes with challenges such as higher implementation cost, consent revocation, and data deletion guarantee. (See the section “Retraction of bodily materials and data”.) It mentions that IBM has a consent management solution that provides tools for modeling consent, a repository for storing it, and a data access management component to enforce consent and log enforcement decisions. The paper says that the question how to automate the consent and manage it efficiently in the interest of legislation, patient autonomy, cost, and data analytics, is still an open problem.^45^

Lastly, with respect to open consent in particular, one paper argues that, because environmental health researchers are increasingly encouraged to share biomonitoring data to create a large, publicly accessible and collaborative research resource, they should consider asking for open consent, in which participants acknowledge and agree to the potential risk of reidentification. The paper argues that this option could become a viable and novel strategy for direct participant engagement in the scientific enterprise through voluntary and open sharing of data and collaborative interpretation of exposure results.^146^

### Communication of results to study participants

Should researchers communicate their results to research participants at all? Two papers note that there is a (well-established) requirement to return results only when participants have accepted to receive results through an informed consent process.^38,140^ Another paper says that, although it is generally accepted that study results should be communicated to participants, there is no agreement within the scientific community about what type of information to relay and how it should be done.^147^

A number of papers stress the value of thinking about the communication of results upfront. Two papers argue that the expectations for exposure assessment or biomonitoring studies need to be set before commencing data collection and setting up results communication protocols.^148,149^ Another paper argues that modern advances in environmental biomarker technology precede knowledge of how to address unexpected findings and that therefore researchers need to design a thoughtful communication plan at the outset of a study and articulate it in the informed consent process.^146^

Five papers comment on the role of researchers with respect to what participants expect from their results. The first paper argues that the decision to participate in omics research may vary depending on what each person believes to be valuable to them, so researchers need to take this fact into account as a part of adhering to the principle of respect for persons.^65^ The second paper argues that researchers may not give participants false expectations or pressure them because that would be patently dishonest and unethical.^87^ The third paper mentions that biomonitoring may blur the distinction between environmental and lifestyle risks in the minds of participants, causing them to feel responsible for exposures that are not part of their lifestyle.^150^ (See the sections “Exposomic and genomic responsibility” and “Ethical guidance for individuals”, especially its subsection “Exposomic responsibility without direct individual control”.) The fourth paper argues that, because public, commercial and institutional dissemination of epigenetics include exaggerated and premature claims about health risks, we need to clearly communicate about what the risks actually are.^151^ The fifth paper argues that biomonitoring participant perceptions of chemical exposure reduction that requires collective action are unclear. The paper argues that their focus group study highlights opportunities to shift responsibility from individuals to policymakers. For example, the paper notes, researchers can provide examples of cases where collective action brought about policy change, and suggest ways for participants to engage in collective action.^152^ (See also the section “Exposomic and genomic responsibility”.)

Three papers stress the costs of communicating results to research participants. The first paper notes that, because responsible report-back is expensive, unintended harm may be created from the use of resources that would otherwise be spent on health or services.^88^ The second paper similarly notes that the cost of both effort and dollars of reporting results back to study participants should not be underestimated.^153^ Anticipating the costs of such efforts, the third paper claims that involving communities at the outset of a study saves time in explaining study results.^82^

There are also a number of papers that provide more concrete advice or lists for projects that want to return results to participants. Five papers outline several communication strategies/approaches used by biomonitoring or environmental exposure studies to communicate results to participants.^147,149,152,154,155^ Another paper argues that the debate concerning the communication of human biomonitoring results has been dominated by researcher perspectives on the issue, which overlooks participant perspectives. The paper presents results from follow-up interviews with participants of a biomonitoring study, on how to communicate individual results in a responsible and meaningful way. The paper provides recommendations for report-back practices based on these results.^156^ One paper provides a list of points to consider when setting up procedures for returning results within epigenetic research.^140^ Another paper argues extensively for the value of a community-based participatory research approach to the reporting of individual results and outlines recommendations for the usage of this approach for research teams.^155^ One paper provides a wider view on “return of results” debates by contrasting discussions about the return of personal exposure results in environmental health research to similar debates in neuroimaging and genetics.^146^ Another paper argues that, when communicating results and recommending exposure reduction strategies, placing target compounds on a graph that relates certainty about health effects of compounds to certainty about how to reduce exposure helps to clarify responsible communication and actionability. Also, the paper hypothesizes, doing so may motivate researchers to articulate what is not known and to work to fill the knowledge gaps.^88^ Lastly, one paper compares participants who initially received individual and aggregate biomonitoring results in an environmental exposure study to participants who initially only received aggregate results with respect to whether and how long they viewed their results, and the feelings they reported about receiving results before and after reportback.^157^

Lastly, three papers make general observations about debates concerning the return of results with respect to research on the environmental effects on health. The first paper argues that legal and ethical frameworks for issues such as the return of results inevitably lag behind the rapidly advancing technological aspects of biomarker research and implementation. However, the authors argue, such frameworks are important for personalized medicine and biomarkers research.^158^ The second paper argues that debates on communicating biomonitoring data to participants should use a broader notion of ethics that considers how ethical responsibility for exposure reduction/protection is passed on to individuals/consumers whose choices can be not just constrained, but also stratified.^150^ (See also the sections “Exposomic and genomic responsibility” and “Ethical guidance for individuals”.)

#### What categories/types of results should be reported?

Given that we should communicate results with research participants, what categories or types of results should we report to participants? Four papers argue the following: the clinical medicine model of full disclosure does not require that all results are communicated but only those that raise the possibility of the need for action. This supposedly leaves out, eg, precautionary action by participants. It is argued that community-based participatory research approaches, approaches that focus on population-level benefits or citizen science “data judo” allow for a much broader report-back of results that are paired with associated benefits.^88,148,149,155^ One paper argues that health-related epigenetic research results that go beyond strict definitions of clinical utility could be returned to study participants.^140^ Similarly, another paper argues that reporting results to study participants may have benefits outside of clinical care, as participants can be given the opportunity to learn about the strengths and weaknesses of science in order to make their own decisions about their results. They argue that this can include, eg, the reduction of exposures as a precaution or becoming engaged in public discourse about chemical use and regulation.^155^ Lastly, one paper argues that researchers need to consider returning results that have personal utility or value to participants, such as results that are emotionally, cognitively, behaviorally, or societally valuable. As examples of each category, they mention relief of anxiety about disease aspects, information that explains a symptom, useful information for reproductive planning, and participating in the discovery of information that might benefit others.^65^

Research results also include secondary, unanticipated, or incidental findings (such adjectives are often used synonymously). With respect to such results, four papers note that genomic and other “omic” or “broad band’ technologies give rise to the issue of returning incidental findings (whether clinically significant or not), which is an area of active discussion in the medical genetics community.^65,159–161^ Within the context of biomarker trials, one paper argues that patients require extensive counseling to understand which unrelated conditions could turn up in incidental genetic findings. The paper argues that this problematizes the ability of patients to give informed consent for choosing either to receive information about incidental findings, or not to receive such information.^161^ Two papers argue that ethical issues related to the disclosure of incidental findings or the return of results need to take into account the cognitive capacity of research participants, eg in situations where the participant is a child.^65,140^ One paper notes the value of multiomics research for communicating incidental findings by arguing that the integration of omics data and family history information with full-genome sequencing can improve clinical decision making about incidental findings.^162^ In the context of setting up a particular multiomics study, one paper summarizes arguments for and against disclosing incidental findings to participants, such as arguments on whether disclosing such information harms or benefits the participants.^163^

#### Reporting results in light of scientific uncertainty or the lack of standards

Many papers also discuss issues with communicating about results when there is lack of knowledge about reference values and health risks. Six papers note that, without the establishment of clear health risks, whether or how to communicate exposures or potential health effects arising from exposures measured in research is one (of the greatest) challenge(s) for scientists.^14,75,88,147–149^ Two articles claim that there is an increasing recognition from the (genetics) literature and international ethics guidance that clinically valid and actionable individual results should be offered to participants. However, they note that the definitions of “clinically valid” and “actionable” are not yet as well established within omics/epigenetics, as they are in genetics.^65,140^ Another article highlights the importance of this topic by discussing how concerns related to the scientific uncertainty about the relevance of exposure results for health outcomes, and the ability to characterize typical exposures, affects the views of researchers and IRBs about the issue whether and how to report back results to participants.^164^

With respect to the uncertainty of results, one papers notes that uncertainty is important to ethical concerns, eg when onetime assessments may not be representative of exposure to some chemicals, which is a limitation that should be explained.^155^ Another article argues that we need to group chemicals into two groups: a first group for those exposures for which there is credible evidence linking exposure with adverse health effects in the human population and a second group for those exposures for which human health risks and intervention levels are unknown. It proposes that biomonitoring results for group one should be communicated together with data on the mean exposure and range of exposure measured in the study. The article argues that results for group two should not be communicated, but retained in the case that health risks are identified in the future and study participants perceive a need to have their previous exposure reevaluated.^147^ Another paper stresses the value of clear communication about results to participants by pointing out that terms like “precision medicine” can be taken to imply an unrealistic level of certainty for treatment decisions.^160^ One paper argues that providing research participants access to their individual biomonitoring results can lead to conflicts between the ethical principles of beneficence, maleficence, and autonomy. They argue that, although an understanding of environmental chemicals may lead to behavioral precautions, uncertainty about the health implications of certain chemicals may cause psychological or financial harm; whereas not sharing results can conflict with the individual’s right to know.^149^ Lastly, one paper argues that disagreement surrounding the question whether or not to provide study participants with data that has unknown health implications can be explained not just by the fact that people have different interpretations of bioethical principles. More deeply, the article argues, people disagree about fundamental different ways of evaluating the meaning and significance of biomonitoring data. The author provides an outline of three different ways that people evaluate the meaning and significance of biomonitoring data differently on the basis of a qualitative sociological study of the history and the contemporary politics of human biomonitoring in the United States. The author argues that their results suggest that resolving debates about the disclosure of results will likely require greater consensus on the meaning and utility/usefulness of data, because these factors shape people’s positions on the value of communicating data.^165^

If there is a lack of standards, are there other informative contrasts that can be provided to participants to compare their results to? One paper argues that participants may be able to learn about significant group risks, if these are provided. However, without risk functions that calculate individual risk, the paper argues, no meaningful individual information can be obtained. The authors point out that this is something that needs to be communicated clearly to participants prior to their participation and reinforced during the explanation of the results.^87^ Another paper argues that comparisons of individual exposure results to a representative sample of their country’s population can lead to a normalization of problematic contaminant levels or cause people to mistake the exposure distribution of the population as a safety benchmark. The authors argue that this could give people a false sense of security or unnecessary concerns when their exposure levels are comparatively high.^88^ Another paper argues that, to better engage the interest of community members, individual exposure results need to be compared to community data rather than more abstract population-level data.^148^ Three papers argue that, if individual exposure results lack clear standards, providing study participants with their individual exposure results in the absence of information about the health significance of the results could cause negative effects such as anxiety and stigma among study participants, legal and economic complications, or the promotion of unnecessary or counterproductive interventions.^88,147,148^

#### Exposome and genome/genetic counseling

Within the literature on genomics, there is a discussion on the value and role of genome or genetic counseling for the benefit of study participants, patients, or healthcare professionals. Is there a need for exposome counseling? From the group of articles that we have included, two papers argue that, if an exposure result is above an established exposure level or a level for which medical intervention is warranted, then further information should be provided to guide the study participant to take appropriate action.^88,147^ Similarly, another paper argues that risks need to be explained in a way that provides people with the information they need to determine appropriate action on the individual and community level.^82^ In the context of personalized medicine and gene × environment risks, one paper argues on functional, ethical, and financial grounds for the value of genetic counselors for clinicians and patients.^166^ Another article mentions that, in the context of clinical epigenetics, there should be a predetermined procedure to transfer epigenetic data to a genetic counselor to help explain the results, especially if the epigenetic data contains actionable information.^118^

Exposome research can require participants to wear or actively use smart sensors that continuously measure exposures. Knowing that these devices are continuously performing measurements, participants might behave differently than usual. One paper mentions that, in the context of occupational health monitoring, one ethical issue that requires attention is the need for counseling and support for individuals who experience stress in monitoring situations.^105^

#### The right to know

Many papers comment on the issue of return of results via the participant’s “right to know” research results that pertain to them. Two papers comment on the justification of this right, arguing that the principle/idea of autonomy includes the right to know as a basis for self-determination in acting on research results.^88,155^ Relatedly, one of these two papers argues that part of exercising a “right-to-know” ethic means that researchers offer participants the opportunity to receive their individual exposure results.^88^ One paper comments that biomarker assessment facilitates people’s right to know what chemicals there are in their body.^39^ However, one paper mentions that there are also costs involved with the “right to know”. They argue that researchers are not expected to actively search for all clinically relevant and actionable individual results because this would unduly burden them; unless this is part of their standard research practice.^140^

Six papers mention that the information provided through the right to know needs to be correct, understandable and avoid raising unnecessary alarm. However, they mention that these three elements are all difficult to execute upon.^14,30,141,148,167,168^ Similarly, one paper mentions that, although participants have a right of information on the health data that are collected, researchers should have appropriate communication strategies to avoid raising panic and inducing behavior that increases risk through other mechanisms.^167^ Another paper says that, even though the European Union provides research participants with a legal right to know, many human biomonitoring studies do not provide individual results on the basis of five different arguments: the lack of relevance of results on the individual level, too limited time and/or resources, fear of causing (unnecessary alarm), scientific uncertainty, or the lack of potential for remediation. They say that these arguments need to be considered when setting up communication strategies.^30^ Taking an overview of the debate, one paper argues that discussions surrounding the right to know have spawned two perspectives. The first perspective favors giving participants the option of receiving individuallevel exposure data to empower individuals and communities to protect their health and participate in policy debates. The second perspective argues that providing individual biomonitoring data is far more likely to cause mental anguish and distress given the absence of health-based interpretations of data. The author reviews the literature on this issue and draws on qualitative interviews to see whether these predictions actually map onto the experiences of individuals who have received personal biomonitoring data.^150^

Three papers discuss the relationship between participants who have exercised their “right to know” and their ability to act on this information. The first paper mentions that, if participants have a right to know their exposure results, yet lack the resources to reduce exposures, then there is a tension between the right to know and their ability or right to act to protect their health.^146^ The second paper argues that their literature research and interviews with scientists and participants suggest that reporting back exposure monitoring results necessitates addressing the rights of study participants to information before, during and after studies, so that participants can make informed decisions and are empowered to take action.^148^ The third paper mentions that, to support the right-to-know while scientific knowledge about health outcomes and dose–response relationships unfolds, environmental researchers advocate for report-back within a precautionary framework. They argue that report-back aligns with the precautionary principle when participants can act on suggestive evidence of harm to human health by reducing preventable exposures.^164^

Several papers discuss potential conflicts between the “right to know” and other values. Three papers question what we should do in cases where the right (not) to know conflicts with privacy, confidentiality or the duty to inform the participant about the need for preventive or curative action when the participant’s exposures are too high.^30,104,169^ Another paper argues that healthy participants in epigenetic research should be given the option to be recontacted when they can benefit from information generated by new risk assessment tools.^170^ One paper mentions that the right to know or not to know may entail close relatives. They mention an example where occupational pollutants affect offspring.^30^ (See also the section “Exposomic and genomic responsibility”.) Relatedly, one paper questions whether exposomics needs to fundamentally rethink ethics, because the return of results that include pollutants found in personal environments and biological samples relates not just to individual ethical ideas, but also social and collective ideas.^171^

## Theme #5: Consequences of research products

This theme concerns itself with the consequences of the products of exposome research for various domains of human activity.

### Anticipation of ethical and societal impacts

How can we anticipate the ethical and societal impacts that exposome research might have? One paper uses a SWOT (strengths, weaknesses, opportunities, threats) analysis to map out a bioscience ethics perspective on biomedicine in the context of the increasing use of omics technologies, biomaterials, high-throughput technologies, and big data for achieving a more holistic and personalized view of health and disease. It mentions that such an analysis is a strategic planning technique for businesses to differentiate between beneficial or risky issues in the context of further developing the enterprise. The paper argues that this analysis allows one to structure a theme into a clear matrix and distinguish between positives and negatives at one glimpse, which a list of advantages and disadvantages does not allow for. On the other hand, the paper argues, this type of analysis does not prioritize any elements within each of the four categories and introduces an element of subjectivity because factors are identified and selected according to one’s practical experiences in the field.^26^ Within the context of epigenetics, one paper argues that a reasonable analytical starting point to anticipate broader societal implications of scientific discoveries is determining how the discoveries compare with existing science. It argues that substantial similarities likely lead to comparable ethical and legal analyses, while extraordinary differences may require a new analytical framework and approach to ethics and law. The paper proceeds to analyze whether the distinctive features of epigenetics, as a matter of ethics and law, differ enough from genetics as to be considered separate from genetics.^130^ Against this idea, another paper argues that important nuances in the nature of the epigenome may be underestimated in normative enquiries, especially when attention remains mainly focused on the differences and similarities between genetics and epigenetics. It argues that such a framing fails to address “biological ambiguities”, which may misconstrue the debate surrounding moral epigenetic responsibilities. The paper goes on to anticipate and present a number of ethically sensitive scenarios, based on scientific nuances in the biology of epigenetic mechanisms, to stimulate reflection on the variety of novel (perceived and real) imperatives seemingly emerging from recent epigenetic findings.^90^ Similar in its approach, another paper sketches a number of explicitly speculative and highly uncertain scenarios to anticipate the societal impact of nanotechnological diagnostics. It argues that such anticipation provides a more robust basis for governance that supports genuine healthcare process than attempts to offset public concerns about controversial emerging technologies via expert risk assurances.^123^ Another paper argues that the combination of large datasets and novel technologies and omics approaches requires ethical reflection from the onset of an exposome project. The project paper mentions that it employs ethics parallel research to identify and evaluate ethical challenges raised during its research project to help realize ethics-by-design and anticipate and integrate ethical norms and societal values in exposome research.^85^

Three papers comment on the way in which ethics research relates itself to natural science. The first paper argues that scholarship on the ethical, legal, and societal implications (ELSI) of epigenetics currently focuses on many hypothetical issues that hype epigenetic ELSI findings in spheres such as the lay media. Its authors claim that this could cause an unwarranted backlash against epigenetic research. Furthermore, they argue that researchers should not abstain from investigating hypothetical issues, but that researchers should spend more time addressing more tangible ELSI issues, such as the question of whether this complex field of study is being introduced to participants, the public, and the media in an appropriate manner.^172^ The second paper argues that we need to reconceptualize and consider the precautionary approach (in contradistinction to the precautionary principle) to guide the cellular biotechnologies with the largest capacity for harm at the individual, group, social, and environmental levels.^11^ Lastly, the third paper makes a general comment on the utility of ethics by claiming that normative argumentation is relevant to the value of biomonitoring for environmental exposures because values are outside the realm of scientific inquiry.^173^

On a critical note, one interview paper argues that, although the “ethical turn” in the philosophy of technology implicitly vows contentment with neoliberal capitalism, the absolute rule of the market, and corporate state-control of what is called “innovation”, there is simply no funding for genuine philosophical work on more fundamental issues such as those pertaining to the very nature and limits of artificial intelligence, of life in the age of biotechnology, etc.^174^

With respect to anticipating how research impacts the public’s understanding of research, one paper argues that the scientific community has an ethical obligation to promote public understanding of the implications of biotech and its risks, harms, and uncertainties. It goes on to say that scientists and other concerned parties should resist pressure to overly promote or exaggerate the impacts of their work, and responsibly communicate and interpret scientific findings and their implications to the public.^11^ Relatedly, one paper argues that, contrary to public-deficit models, it is the case that public ambivalence and concerns with respect to new and emerging technologies are not simply based on a lack of understanding of the science, as they can be tied to issues of trust, values, and experience.^123^ Another paper argues that published research on the ELSI of metabolomics is essentially nonexistent and that one of the reasons why addressing such ELSI are required is because such research is paradigm shifting in science and life changing in medicine and society. It notes that such research can suffer more from failures, if fear of research reduces participation by individuals, groups and nations.^81^ One paper mentions that artificial intelligence systems that work with genomic-, proteomic-, metabolomic-, and dental-specific data to facilitate optimized and personalized treatment strategies and risk management have the disruptive potential to be misused by fraudsters that spread misinformation on social media regarding self-treatment and auto-medication for oral diseases.^57^

### Public health and the push towards more precise and personalized health knowledge

Exposome research increases our knowledge of the effects of the environment on our health at the molecular level. As a corollary, it provides ever-stronger evidence of the relationship between exposures and human health. This increase of the quality of evidence affects the basis of public health policies, because such evidence is part of the basis for engaging in public policy that relates to environmental exposures. In this section, we present the ethical aspects that connect the increase in knowledge generated by exposome research to public health. However, because of the positive relationship between the increased epistemic value of exposome research and the actionability of exposome research findings and products, note that many of the comments and arguments presented here are applicable to other ethical aspects as well.

How does exposome research relate to public health agencies and government generally? Three papers make claims about public health on such a general level. The first paper comments on the role of public health agencies, arguing that they are expected to provide surveillance of known hazards and also identify newly emerging hazards to assure that society can promptly address such hazards.^82^ The second paper argues that, although innovative knowledge may lead to the creation of new technologies that hold the potential to contribute to human well-being, it is not without risks. The authors argue that the assessment and regulation of those risks are ultimately seen to be the responsibility of national governments, often under the stewardship of international regulatory authorities. They claim that scientific standards are an essential component in regulation and risk assessment, but are not impartial actors in knowledge transfer, as such standards actively mediate and effect the creation of knowledge.^9^ The third paper argues that we need to develop regulatory frameworks for personalized medicine; not just for building public trust in new technologies, but also for addressing commercial uncertainty by demonstrating regulatory competence in evaluating the new technologies and creating clear paths to market approval.^175^

Several papers discuss the value of epistemic advances in research for public health in a more general way. One paper argues that, because susceptibility markers are only a statistical indicator whose predictive value depends on the frequency with which those with that marker develop the expected disorder, scientific uncertainty limits our ability to easily determine the existence and nature of sensitive subgroups that we wish to protect via public health policy and environmental laws and regulations.^87^ Relatedly, one paper argues that, in contrast to environmental monitoring, the value of biomonitoring lies in improving our knowledge of the actual levels of chemicals in people (rather than predicting them), decreasing the uncertainty associated with assessing human risk and vastly improving the ability to make timely and appropriate public health decisions and regulations.^39^ Similarly, one paper argues that, via what they call “molecularization”, problems of environmental pollution that have a significant impact on population health may lead to interventions that are different from those that would follow from focusing on individual susceptibility to such pollutants. They also argue that, because epigenetics can be used to identify and measure within the body the effects of pollutants from outside the body, epigenetics transforms external determinants of health into internal ones.^176^ (See also the section “Molecular redefinition of diseases”.) One paper argues that we need funds to establish public health centers that can proactively respond to problematic results uncovered during biomonitoring research, particularly when community action is needed. It also argues there is a related need for adequate environmental health training among health professionals, as they often lack such training.^164^

#### Public health practices

A number of papers mention a number of ways in which scientific advances can affect public health practices. One paper argues that, through an integration of different levels of biological, social, environmental, and behavioral complexity and the unbiased study of population-based samples with broad statistical approaches that include bioinformatics analyses, epidemiology can develop a wide public health/population-based framework for systems medicine.^177^ Another paper focuses on the resolution of public health interventions by arguing that, regardless of the laudable goal of public health to address the entire population, this view also risks treating communities as homogenous organisms, overlooking the diverse needs of distinct subpopulations defined both by variations in biology and environment.^178^ Similarly, one paper argues that precision medicine allows for the integration of social determinants of health with a greater understanding of the dynamic interplay between biological, behavioral, social, and environmental risk factors, and protective factors experienced across the life course. The paper argues that this allows for the possibility of precision public health that can help to fill the substantial gaps left in many areas from the one-size-fits all approach in large-scale untargeted public health or clinical interventions.^179^ However, another paper argues that, because the classical definition of “public health” refers to disease prevention at the population level, it may potentially conflict with the idea of prevention through personalized diets guided by individual genetic make-up.^180^ Similarly, another paper argues that the advent of molecular techniques might cause us to redirect our focus from identifying risks in the exogenous environment to identifying high-risk individuals and then making personalized risk assessments. It argues that this would direct our focus to a form of clinical evaluation, rather than public health epidemiology, which distracts from the important public health goal of creating a less hazardous environment.^181^ (See also the sections “Distinction participant–patient and epidemiology medicine” and “Clinical translation of exposome research”.) One paper provides an overview of complementary definitions of precision public health and summarizes a number of papers that address the idea of precision public health.^182^ In the context of epigenetics, one paper states that epigenetics has been used as an argument for better preventive public health policies, as epigenetics allows for a better mechanistic understanding of the developmental origins of health and disease. However, it goes on to state that some scholars argue that the significance of epigenetics should not be overstated because of a number of reasons: the field is still in a nascent state, there currently is an absence of compelling evidence of epigenetic inheritance in humans and there is a high probability of confounding variables in environmental and social epigenetics studies. Also, it states that there are meta-ethical questions that need to be answered, which relate to the degree of normative-prescriptive value that should be granted to empirical findings.^91^ Relatedly, another paper argues that defining epigenetic responsibility as *a priori* prospective and belonging mainly to the government (as some have done in the literature) is misleading because it is simplistic, ineffective, and ethically problematic. The paper claims that there is a diversity of types of epigenetic responsibility which will likely emerge from further developments in epigenetics.^90^

#### Public health and reference values

A number of papers discuss the relationship between scientific evidence, reference values and the supposed role of the government in protecting public health. One paper argues that the government should decide whether it is willing to take remedial action only when there is evidence of significant exposure, or also when a community biomonitoring study does not reach statistical significance or high power due to problems such as low-dose exposures.^105^ Another paper argues that human biomonitoring enables the development and re-evaluation of national reference values and checking of possible exceedance of health-related limit values, as it improves our knowledge of causal links between environmental factors and health. It argues that human biomonitoring can thus support the surveillance of the efficiency of political risk reduction measures.^183^ Similarly, another paper says that regulatory agencies set safe exposure limits, which implies that environmental exposure at levels below these limits are not of health concern, even when such exposure leads to detectable concentrations in the body as detected by sophisticated biomonitoring methods. The authors note that this view is being challenged by results from studies on animals which reveal low-dose effects of endocrine toxic chemicals.^147^ In the context of epigenetics and environmental regulation, another paper argues that, because environmental laws in the United States were generally written to protect the public from certain hazardous exposures rather than certain health effects, the laws apply to all human health effects, including epigenetic harms. The difficult regulatory question concerns when the scientific evidence is sufficient to warrant regulatory action to prevent epigenetic harms.^130^ (See also the section “Law and international treaties”.)

#### Public trust in regulation

We found three papers that relate the issue of public trust to regulatory processes. In the context of personalized medicine and the usage of omic type data, the first paper claims that bioethics committees have emerged as brokers of public trust in the postgenomic regulatory domain.^175^ The second paper claims that public accountability and trust in a regulatory system are best cultivated in an environment of participation and transparency.^31^ The third paper claims that, to proactively build public trust in new omics fields, it could be timely to create a publicly funded multidisciplinary oversight body that carries out independent, impartial, transparent, and integrated innovation analyses and prospective technology assessments across the omics fields.^184^

#### Researcher engagement with public health and the public

Two papers discuss what researchers should do to protect public health with their findings. The first paper asks a number of analytical questions on what researchers should do when they encounter high levels of a substance such as polybrominated diphenyl ether in food products when there is a lack of clear regulatory guidance. The author warns that reporting results tied to a specific product or manufacturer may also lead to an injunction against publication or the journal that publishes the paper.^185^ The second paper argues that citizens, but especially scientists and physicians, have a justice-based duty to protect children from developmental toxicity.^186^

Two other papers comment on the ethical aspects of scientists who engage in policymaking. The first paper argues that scientists who engage in policymaking can potentially undermine scientific authority because, during policymaking, scientists have to make value judgments and are challenged by scientific uncertainty. The paper argues that this blurs the line between science and policymaking, and prompts the question: Should scientists push for policy based on results from their biomonitoring studies? The authors argue that this issue raises further ethical dilemmas concerning scientific objectivity, credibility, and involvement in the regulatory or legislative spheres.^14^ In the context of biomonitoring and environmental exposure research, the second paper reports that, although there was little reluctance among researchers to suggest individual behavior changes to reduce exposures, the appropriate role and capacity of researchers to advance and support collective action and policy advocacy was controversial. It also reports that, whereas some researchers assisted study participants and communities in responding to exposures results, others expressed concern that this would compromise the integrity of the research or cited a lack of ability or legal authority.^164^ (See also the section “Exposomic and genomic responsibility”.)

Lastly, one paper argues that human microbiome researchers should reflect on how the outcome of their R&D could benefit society at large because there is a need to raise awareness about the value of human microbiome R&D, to educate the public with accurate scientific evidence, and to combat misinformation.^113^

### Distributive justice

“Distributive” justice concerns itself with the question: What is a morally justified distribution of benefits and burdens among members of society?^187^ Benefits and burdens can be various things, such as material resources, services rendered or required, or disease burden. In its most simple form, someone who wishes to achieve distributive justice would advocate for an equal allocation of benefits and burdens among the public. Such a person would be a “strict egalitarian” and view any of sign of inequality between (groups of) people as an injustice that needs to be rectified. However, the principles for determining a “just distribution” can also be more complex. Rawls, who is also an egalitarian, advocates for the so-called “Difference Principle”, which holds that society may justly diverge from an equal allocation of benefits and burdens “if that would make the least advantaged in society better off than they would be under strict equality”.^187^ Due to their focus on distributions of goods among the population, egalitarians do not primarily focus on the individual’s health, but on relative differences between people’s health, ie, inequalities. We structure this section via often-used concepts in distributive justice: inequality, equity, environmental justice, and intergenerational justice, as the papers that we analyzed almost never specify which kind of egalitarian principles they adhere to.

Two papers comment in general terms on the usage of epigenetics in discussions on distributive justice. The first paper argues that the dynamic nature of the epigenome and its variable sensitivity toward change in numerous phenomena adds more complexity to the assessment of health inequalities and demands a more inclusive concept of health when used in discussions of inequities.^188^ The second paper argues that discussions of distributive justice in epigenetics need to consider the diversity and complexity of epigenetic mechanisms, as these complicate whether certain types of risk or disease are fixed or develop over the course of life, or are in the domain of chance or choice. The authors argue that each of these complications is key considerations and distinctions for various egalitarian theories.^90^

#### Inequality

Four papers comment on inequality as such. The first paper argues that a risk for public health is that new technologies and tests catalyzed by personalized medicine might result in increased social deviation between those who can afford them and those who cannot.^17^ The second paper argues that tests and treatments for reversible epigenetic alternations are likely expensive and thus epigenetic discoveries could lead to an increase in health inequality.^130^ The third paper argues generally that environmental epigenomics and epigenetic epidemiology can be leveraged to manage social inequalities and external determinants of health through public policies.^176^ The fourth paper argues that, although the full realization of proteomic preventive profiling lies well in the future, it has significant potential to advance biomedical knowledge and health, and reduce inequality in access to health care.^20^

#### Equity

Several papers comment on the idea of equity. The first paper argues that exposome research presents an opportunity to query the specifics of health inequities such as structural racism.^189^ The second paper mentions that, if applied inequitably, precision medicine technologies and approaches have the potential to actually worsen population disparities.^179^ The third paper argues that precision health will only be valuable if it can be advanced equitably. The paper goes on to argue that, for precision health to be valuable, it must be prioritized among underresourced settings for underserved, marginalized, and rural populations. The paper argues that precision health needs to be conducted with historically marginalized communities first, instead of high resource settings, because otherwise, it is quite likely that precision health will exacerbate inequities.^32^ Similarly, one paper recommends more precision medicine biomarkers research and funding in support of neglected or understudied populations worldwide for ethical and inclusive representation in global science.^190^ Relatedly, one paper argues that, on the global scale, the development of nanotechnological diagnostics and takes place in the wealthier parts of the words, resulting in the so-called “nano divide”. It argues that countries most in need of good health care may not be able to afford nanotechnological diagnostics, and developments in the field may be biased toward diseases quite common in the western world, leaving important and perhaps more urgent diseases unaddressed. The authors mention that equity issues are also possible on the national scale, either due to similar dynamics at the local/regional scale or due to “orphan populations” for whom existing treatments are ineffective or too risky due to unique genetic dispositions.^123^ Another paper argues that omics and digital healthcare technology can be used to reduce health disparities for forcibly displaced individuals, and presents an “ethical plan” for doing so.^191^ On a critical and applied note, one paper argues that, because structural inequities may persist within families across generations, this raises concerns that epigenetic marks may be erroneously considered heritable if inequities are not adequately captured or measured.^110^

Intergenerational equity is also discussed. One paper mentions that intergenerational equity refers to the obligation of each generation to serve as a steward of the planet, its environment and its myriad species of plants and animals. It goes on to argue that transgenerational effects of hazardous exposures via epigenetic-mediated processes affect future generations. However, the paper argues, it is unclear how to translate this idea into an environmental ethos of minimizing toxic exposures and the harms that they cause.^130^ Another paper argues extensively that evidence on the intergenerational epigenetic programming of disease risk can broaden the scope of public health preventive interventions to include future generations as long as these generations overlap, by appealing to various distributive justice principles.^192^

#### Environmental justice

On the topic of environmental justice, one paper argues that we need to separate environmental justice from environmental equity because environmental equity makes it sound as if, when we all share the problem, that is okay.^82^ We assume that the authors intend to distinguish their view of environmental justice from a “strict egalitarian” view. Another paper connects biomonitoring to the issue of environmental justice. It argues that environmental justice advocates have approached biomonitoring with caution because of concerns that “after the fact” measurements cast communities as environmental hazard detectors, and because biomonitoring can potentially “overscientize” environmental health problems due to the overlooking of upstream causes that are rooted in social inequality, economic exploitation, and racial discrimination. The paper also states that marginalized communities can use biomonitoring to record the extent of communityspecific contamination and leverage government funding, industry action, or legal remedies.^148^ One paper asks the question of what the duties and responsibilities are of government officials, environmental scientists, and epidemiologists who are developing advanced health risk assessment procedures for helping to ensure that the scientific knowledge obtained is used in a way that benefits at-risk individuals and communities without adversely affecting environmental justice concerns.^171^ (See also the sections “Exposomic and genomic responsibility” and “Researcher engagement with public health and the public”.)

We found a number of papers that argue about environmental justice in the context of epigenetics. One paper argues that, because epigenetic effects have been associated with exposure to various toxic chemicals, airborne pollutants, pesticides, and other harmful substances, epigenetics reinforces the need to consider environmental justice issues.^130^ Similarly, another paper argues that epigenetic social justice may require that we provide citizens with a safe environment free of substances that may damage the epigenome.^135^ One paper argues that epigenetic testing raises questions about the perpetration of socioeconomic disadvantages to those who are more likely to be exposed to dangerous environments. The author argues that the requirements of environmental justice suggest an ethical and social obligation to prevent epigenetic damage where such prevention is practicable and economically feasible.^125^ One paper reports that epigenetics has been used as a tool to help claim that unfair health disparities could and arguably should be prevented through social policy.^91^

The issue of environmental justice comes paired with the so-called “general environmental justice hypothesis”. This hypothesis is related to the topic of the social determinants of health. As one paper explains, this hypothesis holds that people of lower socioeconomic strata are more exposed to environmental pollution than people of higher social strata. The authors tested this hypothesis in the case of Flemish adolescents and found that the association between socioeconomic status and internal bodily concentration of exposure to environmental pollutants is more complex than can be assumed based on this hypothesis. It argues that, depending on the (type of) pollutant, adolescents with a lower socioeconomic status have either higher or lower internal concentrations than adolescents with a higher socioeconomic status. The authors note that environmental injustice has been shown to happen in different ways and that not finding consistent negative social gradients in external exposure does not mean that inequality in later health effects will not arise from it.^193^ Another paper takes a broad perspective on this hypothesis and claims that multiple studies support the idea that the traditional environmental justice hypothesis is not always in line with the results of human biomonitoring studies, as social differences have an effect in both directions (low as well as high).^79^ In the context of metabolomics, one paper argues that claims based on metabolomics need careful review and include issues of social justice, racial disparities, and privacy because built environments in more impoverished areas are likely to provide tangible evidence of disproportionate environmental toxins relative to more influent areas.^81^

### Discrimination

In this section, we present the general comments on the issue of discrimination that we found in the literature. The comments that were made about discrimination related to a particular part of the research, such as “exposomic responsibility”, bias, or non-discrimination law, have been incorporated in other sections of this paper.

Two papers discuss the potential consequences of improved and new health risk assessments. The first paper asks several questions about what the potential ethical, legal, and social implications are of developing improved health risk assessments that leverage emerging findings from epigenomics, exposomics, and genomics. The paper asks: Will people who live in at-risk locations and already face great personal and community-wide challenges become stigmatized by such assessments and face increased discrimination from financial institutions or other societal groups/institutions, or will their health improve and will this increase their employment and educational opportunities and access to health care?^171^ The second paper argues that the use of proteomic testing rather than gene analysis may even reduce the risk of individual “genetic” discrimination.^194^

Many different papers argue or warn about the potential discriminatory usage of exposome-related information or technology. One paper generally warns of the use of omic data for discrimination by employers or insurance companies.^142^ More specifically, another paper argues that inherent in the use of genetic and epigenetic data to stratify patients into groups and subgroups is the risk of stigmatization and discrimination at the individual, community, and population levels.^195^ Similarly, one paper warns of the use of more exact characterizations of the migraine population in treatment trials as a basis for treatment limitations and denials by insurance and other third-party payers.^48^ Another paper warns for the ability of plasma proteomes to reveal personally sensitive information that can be used to discriminate against people.^38^ With respect to the usage of smart sensors, one paper warns for the use of personal real-time monitoring information by third parties, which can have consequences for one’s insurance (such as biochemical discrimination) or personal life.^123^ Another paper warns that the identification of specific environmental exposures could worsen discriminatory practices, such as “redlining” practices in neighborhoods with lead contamination.^82^ Two papers comment on the discriminatory potential of tests. The first paper mentions that moral stigma and discrimination can come paired with the results of epigenetic testing.^125^ The second paper warns for the usage of biomarker tests to discriminate in job placements, insurance, and acceptability for loans (such as when they test for demographic characteristics like race or ethnicity that have been historically discriminated against).^87^

Three papers comment on the potential discrimination of healthy people through the usage of predictive technology. The first paper mentions the possible use of immune biomarker results for discrimination against otherwise healthy individuals by insurance companies or potential employers.^158^ The second paper argues that if the biomarkers for psychopathy were reliably detectable at an early prodromal stage or prior to onset, the issue of who should have the authority to collect or access this potentially stigmatizing data is a complex concern.^196^ The third paper mentions that employers, insurers, and others with an interest in the future health of an individual are under no present legal constraints that would prohibit asking for epigenetic information that could reveal potential health problems in the future. It argues that such epigenetic information could be used to discriminate against individuals who have not yet developed and may never develop an illness.^135^

Three papers relate discrimination to the issue of “racialization”. The first paper asks the question whether a new and greater epigenetic understanding of health disparities will help target interventions or give rise to increased racialization without structural determinants of inequities being addressed.^110^ The second paper provides a discussion of both epigenetic and environmental racialization.^197^ Lastly, the third paper reports on discussions of discrimination in epigenetics, such as the reification of biological races, the reinforcement of stereotypes when measuring and discussing social disparities using biological metrics, and the normalization of privileged bodies.^91^

### Law and international treaties

#### Environmental and reproductive tort law

Four papers relate the increase in evidence brought about by research to environmental and/or reproductive tort law. The first paper asks the question whether epigenetic data can be used to support claims of negligent parenting.^110^ The second paper goes on to argue that epigenetic information does have the potential to influence environmental and reproductive tort law, as it can provide scientific evidence of harmful exposure at the molecular level.^140^ The third paper argues that, as an example, if an increase in lung cancer risk can be attributed to historical cadmium pollution due to industrial activities in a certain region, the population concerned will be alarmed and look for specific actions from policymakers and the respective industry. It goes on to argue that according to the principle that the polluter should pay, that industry should contribute to the sanitation of the contaminated soil and to the evaluation of the effects of their interventions. The authors mention that a human biomonitoring study in the area may be one of the tools to do this.^30^ The fourth paper reports that some argue that epigenetics may help to show the causation required to employ population studies as evidence in tort law, by providing information about the molecular mechanisms that link, for instance, exposure to chemicals and the occurrence of diseases. The authors report that this can aid, not just in providing compensation to victims of environmental harms, but also for developing new regulatory schemes and policies that better reflect our understanding of the effects of toxins on the body. The authors continue to report, however, that legal scholars recognize a number of barriers to the usage of such evidence, such as the long latency period between the harmful act and the emergence of symptoms, the lack of access to both the judicial system and the necessary evidence for vulnerable parties, and the difficulty of quantifying epigenetic harm with certainty. However, the authors note, these barriers may be overcome by revising laws, regulations, and policies.^91^

#### Human rights and ethics declarations

Do the findings of exposome research require changes to human rights or ethics declarations to better protect individuals? One paper discusses various human rights declarations that have been adopted to address emerging ethical, legal, and social concerns associated with genetic research and technology. It argues extensively that the emergence of epigenetics and other postgenomic sciences disrupts such declarations in two ways. First, the paper argues that these sciences require us to reformulate some provisions already contained in existing declarations to ensure their applicability to nongenetic biological sciences. Second, the paper argues that these sciences require us to reframe the human rights approach from a focus on the rights of the liberal individual to a more inclusive framing that includes protections against pervasive social and health disparities, environmental harm and injustice, and intergenerational health inequities.^198^ In the context of developing omics analyses and personalized medicine for astronauts, another paper argues that ethical issues go beyond the human experimentation principles in the Declaration of Helsinki on ethics for medical research involving humans, and names issues such as pre-flight screening and whether there is effective informed consent of astronauts given the high number of unknowns in space flight.^199^ (See also the section “Making consent informed”.) One paper argues that, because there is increasing evidence of transgenerational effects of (grand)parental lifestyle choices on children and subsequent generations, we need to better protect children’s health by extending the powers of the United Nations Convention of the Rights of the Child so that it is the basis of all government policy for children and young people. It argues that this declaration should include a statement of intent to make children’s health, education, and well-being the first priority for public policy and to enshrine this in law.^200^

#### Nondiscrimination law

We found several papers that make claims about the future effectiveness of existing laws that aim to protect citizens against genetic discrimination. In particular, the United States’ Genetic Information Nondiscrimination Act (GINA) is often mentioned in the literature. One paper claims that GINA protects Americans against discrimination based on their genetic information and paves the way for people to take full advantage of the promise of personalized health care without fear of discrimination.^95^ Another paper argues that GINA does not, at this time, provide protection for most health- and behavior-associated metabolites. The paper claims that this is a distinct privacy risk from other nongenetic health information because the metabolome is all encompassing, not targeted, and there are no guidelines currently for delineating that which is relevant and reasonable for insurers from that which needs further protection from discrimination.^81^ In the context of epigenetics, one paper argues that, because the wording of GINA strongly suggests that it does not apply to epigenetic information, we probably need an amendment or new legislation to protect against epigenetic discrimination.^130^ Lastly, one paper mentions that many nondiscrimination policies, such as GINA, do not contain any explicit statute prohibiting discrimination based on individual epigenetic information. The authors say that this calls for caution and accountability for direct-to-consumer epigenetic test companies, none of which currently mention potential risks of misuse or absence of legal protections against epigenetic discrimination in their policies. The paper calls for bioethicists, legal scholars and policymakers to reflect on whether the rationale behind genetic nondiscrimination statutes also apply to different epigenetic data types, as well as evaluate and consider the adverse effects that public worries about potential epigenetic discrimination may have on participation in epigenetic research and the eventual uptake of physician-prescribed epigenetic tests.^201^

#### Privacy law

Researchers that work with health data need to comply with the privacy laws that are relevant for their research. In this section, we present the ethical aspects that relate to privacy law.

We found three papers that comment on the European Union’s General Data Protection Regulation (GDPR). The first paper argues that the GDPR has promising attributes for ensuring the protection of personal data that are collected and processed for clinical proteomics, but that the GDPR also has a number of potential adverse impacts on enhancing health data research. For clinical proteomics, there are limitations posed for the collection, processing, and use of data that need to be overcome.^202^ The second paper argues that it has been claimed that, if the GDPR is loosely interpreted, it may lead to the indefinite storage of personal and sensitive data for any research purposes and processed without knowledge of the data subject. Also, the paper mentions that this interpretation may not even provide the data subject with the option to opt out.^203^ Before the GDPR was signed into law, a third paper argues that it is necessary for each participating Member State to obtain ethical approval individually, but that the ideal situation would be that international projects are able to apply for international ethical approval at the European Union level.^104^

Two papers discuss the regulatory burden created by privacy law. The first paper discusses how very strict ethical and data safety protection rules could hamper the establishment of molecular epidemiological studies and biobanks.^141^ The second paper argues that, in the existing ethical framework including national, European, and international regulations, international conventions and declarations, and guidelines and opinions, researchers may be put in situations in which it is unclear how to act in accordance with all necessary legal requirements of the ethical aspects of research. The paper argues that for transnational research projects, which are important for further research on the health impact of environmental factors on a large scale, and in which transfer of sensitive personal data and/or biological samples from one Member State to another is common practice, the labyrinth of rules and guidelines becomes an even larger clew. Consequently, the paper argues that significant scientific developments may be missed whilst juggling with ethical concepts and rules.^30^

We found two papers that comment on the ethical aspects of compliance. The first paper discusses how to comply with privacy regulations in the context of precision health data usage (regulation from the United States, the European Union, and Australia).^45^ The second paper discusses how to render the plasma proteome ethically unproblematic and GDPR compliant.^38^

In the context of post-genomics, one paper surveys various (international) data protection provisions and evaluates the extent to which they are gene-focused and not focused enough on protecting epigenetic and other post-genomic information.^198^

### Clinical translation of exposome research

As it is currently practiced, exposome research is a form of population research. Consequently, exposome research adopts (and aims to improve upon) the concepts, standards, and methods of epidemiological research. But as our understanding of the environment and human health grows in scope and resolution, exposome research can become clinically relevant and require translation from cohort-to-bedside. Thus, there is a need to be sensitive to the differences between exposome research and medical science in the ethical analysis of exposome research. In this section, we group the ethical aspects related to the clinical translation of exposome research.

Three papers provide such comparisons of exposome(-related) research and medical science. In an overview article, the first paper describes the differences between the medical model and the exposure science model in terms of their interpretation and use of biological data, such as risk, dose, and proposed response to elevated mercury levels observed in umbilical cord blood.^89^ Relatedly, the second paper argues that epidemiology can use bioinformatics, omics studies, and systems biology as an opportunity for more successful integrative strategies that can help understand complex diseases and contribute to personalized medicine.^177^ The third paper argues that environmental and occupational exposure data often form little or no part of medical history taking in clinical settings, and thus there is an urgent need for bridging and overcoming silos. It notes that the exposome might achieve the incorporation of environmental health into personalized medicine by integrating individual exposure history, lifestyles, and genetic susceptibilities, even though the exposome approach may gather a lot of measures on exposure without proper concepts and tools to associate these with health risks, address interventions and model exposome change over time. The paper also relates the exposome to the OneHealth approach.^68^ Comparisons such as these lie at the basis of the ethical analysis of the clinical translation of epidemiological research such as exposome research. On a more methodological note, one paper discusses the use and execution of a “consensus conference” as a strategy for translating basic research in a way that overcomes social and technical barriers and includes lay communities in the translation mix. The paper received input from 15 Boston-area residents on ethical, legal, and scientific issues surrounding biomonitoring.^204^

Several papers address the process of clinical translation. One paper argues that, because exposure scientists lack the medical credentials required for assessing patients and conducting clinical exams/tests, clinicians are the default gatekeepers for basic research in exposure science. The authors continue to argue that translating exposure science advances from “bench to the bedside” is important and feasible, but necessitates the combined and sustained effort of exposure scientists and clinicians.^89^ In the context of omics research, one author argues that the proper evaluation of clinical diagnostic tests is of value for commissioners of health services that need to decide which tests should be available, and for companies whose role it is to develop and bring new tests to the market.^19^

In the context of epigenetics, one paper argues that “molecularization” and “biomedicalization” are likely to favor a clinical translation of epigenetics at the expense of a policy translation. Furthermore, they argue that there are four pathways of thinking through which a largely clinical translation of epigenetics could contribute to the further consolidation of the current biopolitical landscape; pathways which they name internalization, isolation, commodification, and technologization.^176^ Another paper on epigenetics reviews the ethical, legal, and societal issues of epigenetic research in personalized medicine.^205^

Three papers discuss barriers to progress in the clinical translation of research. Within the literature on epigenetics, one paper reports on discussions of subtle biases and barriers that may impede the translation of scientific findings into fair and effective health interventions.^91^ (See the section “Bias in data, analysis, algorithms, and artificial intelligence”.) The second paper argues that the lack of standardized assessment and reporting criteria for ethical issues such as informed consent for testing and communication of results to patients are reasons for the lack of translation of big data, AI, machine learning, and omics technologies into the clinic.^162^ (See also the sections “Informed consent” and “Communication of results to study participants”.) The third paper argues that there are several likely barriers that impede progress in clinical translation: that biomarkers are splintered into numerous costly patent-protected tests, that each test requires Food and Drug Administration (FDA) approval and that there is an open question whether insurance companies reimburse biomarker assays.^95^

Four papers comment generally on the ethical aspects of increased stratification of diagnosis and treatments. The first paper argues that, because stratified medicine inherently restricts the potential number of patients for a drug, the development of a biomarker that will promote the usage of specific treatments might exclude a proportion of the currently treated population. The authors note that this may affect commercial revenues for specific therapeutics.^158^ The second paper warns that an ethical issue of personalized medicine is that targeted therapies may have high costs and very poorly improve the survival rate of patients, which prompts the question whether it is morally and socially legitimate to allocate such an amount of financial resources for such a little healthcare benefit.^206^ In the context of precision medicine, the third paper poses the question whether the biomarker-based stratification of patients into groups that are offered different treatments conflicts with the principle of equal treatment. The paper argues that this depends on the properties of the biomarker, but that biomarkers with sufficient analytical validity, clinical validity, and clinical utility may be seen as an ethically relevant factor for giving unequal treatment to patients with the same disease.^207^ The fourth paper warns for the creation of “molecularly unstratified patients” who are not eligible for a targeted therapy, which leaves them out of scientific and technological advances and which challenges our ability to provide equitable access to care for all patients.^208^ Two papers relate such increased stratification to the issue of discrimination. The first paper warns for social discrimination in less privileged groups due to precision medicine’s ability to stratify patients.^209^ The second paper argues that an effort is needed against the possible discrimination in access to treatments that may happen if groups of “nonresponders” in a treatment based on a large number of individuals from various populations are definite minority populations.^210^

With respect to regulatory approval in the context of the United States, three papers mention that it is important to have early communications with the FDA when developing omic tests, eg, due to the FDA’s evolving view of regulatory enforcement discretion for omics-based tests.^23,92,93^ Another paper investigates the current regulatory framework established by the United States’ FDA for precision medicine and identifies challenges and concerns through a study of related literatures.^211^ Relatedly, one paper performed a scoping review that describes the major perceived regulatory, intellectual property, and reimbursement challenges to the development, translation, adoption, and implementation of personalized medicine products into clinical care.^212^

#### Clinical usage of exposome tests and diagnosis models

A number of papers comment on the ethical aspects of the clinical usage of omics tests. One paper argues that associations between omics predictor results and clinical endpoints may establish the clinical validity of a test, but does not always translate into clinically meaningful associations or provide clinically useful information. To establish clinical utility, as opposed to clinical validity, they argue that there must be evidence suggesting that usage of the test is likely to lead to a clinically meaningful benefit to the patient beyond that provided by current standards of care. The authors conclude that clinical trial designs for definitive evaluation of an omics test must therefore begin with a clear statement of the target population and the intended clinical use.^93^ Relatedly, another paper argues that predictive biomarker assays that are being used commercially are not always clinically validated, even though they are analytically validated. They claim that the indirect risk associated with any clinical decision stemming from the inappropriate use or premature adoption of any biomarker test result must be recognized as a risk of the biomarker.^213^ (See also the section “Measurement technologies”.) One paper argues that the goal of adapting omics tests to clinical decision making is to identify, interpret, and report all the medically relevant data. It claims that the two major challenges are: accurate interpretation of massively complex datasets and defining the limits of the technology.^214^ On that topic, one paper argues that, in order to have a high likelihood of translating metabolomics-based biomarkers into a routine clinical test, professional and regulatory agencies should provide updated robust guidelines for study design, data acquisition, and validation from the start of a project. The paper goes on to claim that predictive omics-based tests and fully automated clinical analyzers present a major ethical hurdle, as they will change relationships between patients and healthcare providers, increase physician visits, laboratory tests, and patient anxiety.^210^

In the context of epigenetics, two papers comment on the ethical aspects of commercial direct-to-consumer tests. The first paper argues that most direct-to-consumer epigenetic test companies advertise their tests as providing medically relevant results that consumers can use to improve their health. However, they argue, these tests have not been given regulatory approval by the United States’ FDA or shown unequivocal evidence of clinical utility. The authors proceed to say that the clinical utility of new tests should be more transparently recognized and not downplayed in promotional messages.^201^ The second paper mentions that there is a lack of standards, guidelines, or contractual agreements to inform and regulate the collection, use, and disclosure of epigenetic data that are generated by direct-to-consumer companies and also that there is a lack of reliable and accurate information about this topic for the lay public.^198^ Relatedly, one paper performs a content analysis of websites and policy documents of 12 international companies that sell either direct-to-consumer epigenetic or microbiome tests. The paper raises questions on these companies’ presentation of scientific validity and medical relevance of tests, issues of poor accessibility of policy documents, data sharing and privacy, and risks of secondary and misuse of data. The paper suggests that we should develop best practice standards and regulation for these companies, and calls for more scholarly attention to the rise of multiomic direct-to-consumer products.^112^

Several papers comment on the ethical aspects of using biomarker-based clinical diagnostic models. In the context of personalized immunology, one paper argues that there is a strong need to identify reliable molecular biomarkers for more precise stratification of patients than when physicians base their subjective judgment on clinical symptoms alone.^51^ Another paper argues that models generated from large complex datasets are harder to interpret, and that the task of generating explanations from nonlinear models is nontrivial. However, the authors write, clinicians (rightly) crave actionable insights at the time of decision making that is in line with the “five rights” of decision support (the right information, delivered to the right person, in the right intervention format, through the right channel, and at the right time in the workflow). The authors conclude that generating explanations to interpret results from a model is thus critical for most conditions of interest.^84^ Two papers mention the “black box” problem for artificial intelligence models, which they argue refers to the lack of explicit declarative knowledge representations in machine learning models, which, among other things, makes it hard to provide a lay explanation of how such a model generates its output.^77,84^ In that context, one paper discusses strategies through which we can make AI systems explainable and interpretable, which can help facilitate the right of individuals to be explained the reasons why an algorithm has taken a decision that affects their life.^121^ Lastly, in the context of clinical decision making in precision psychiatry, one paper argues that the usage of AI may pose a special ethical concern due to the significance of human contact in mental health services.^209^

### Exposomic actionability for individuals

#### Ethical guidance for individuals

How should participants think about exposures discovered through exposome research from a first-person perspective? What ethical guidance is provided to them by the literature? How can they make the most of information about exposures with (often negative) health effects? One paper mentions that, to see whether one’s personal exposure to environmental chemicals are safe, individuals can draw upon reports such as the National Report on Human Exposure of the Centers for Disease Control and Prevention (CDC). However, the paper argues that such comparisons can lead to the normalization of problematic contaminant levels or the construal of such levels as safe.^88^ Another paper says that, in postgenomic health care, there is a psychological risk of data overload for patients because data on the molecular processes of a person might not be the right data to comprehensively determine an individual’s state of health and need for an intervention. The paper mentions that, at the same time, when sufficient data about personal bodily functioning are available, this can make individual patients more responsible to manage their own sense of health, empower patients to manage self-care better and, subsequently, ease the economic constraints on the healthcare sector.^121^ One paper argues that epigenetic information can help individuals to identify products that contain dangerous chemicals and reduce their exposure to those chemicals. However, the paper mentions that epigenetic testing could challenge individual autonomy because it might change family dynamics.^125^ Another paper notes that risks for many diseases can be estimated long before therapy is available, which may negatively affect people’s lives if they are acquainted with their risks and do not receive proper counseling or psychological advice.^215^ Similarly, one paper argues that, as greater attention in medicine focuses on individual susceptibility to disease and environmental agents, patients can become inappropriately concerned about avoiding environmental exposures. In an extreme form, so it is argued, this can lead to “environmental anxiety”.^194^

Two papers mention how participating in research can provide individuals with benefits. One paper argues that participating in epigenetic research could and should provide participants with actionable results that can provide clinical or nonclinical health benefits, such as health-related life choices.^140^ Another paper mentions that participating in omics research can provide participants with personal utility, such as results that are emotionally, cognitively, behaviorally, or societally valuable. As examples of each category, the paper mentions relief of anxiety about disease aspects, information that explains a symptom, useful information for reproductive planning, and participating in the discovery of information that might benefit others.^65^

Relatedly, two papers report on ways in which participants of biomonitoring studies act on exposure information. The first paper, in the context of receiving individual results for biomonitoring and environmental exposures, reports that participants were not unduly worried by their results and the associated scientific uncertainties, learned about environmental health, sometimes took steps to reduce exposures, began thinking about thinking about possible sources of chemicals in their bodies and homes, felt respected and grateful and saw their contribution to science in a brighter light, and were more committed to participation.^155^ The second paper argues that it shows that being biomonitored leads individuals to think and speak about themselves in terms of new exposure-related categories, and to see themselves as bearing varying degrees of responsibility for their own past, present, and future exposures, which could cause them to feel responsible for exposures that are not part of their lifestyle.^150^ (See also the next subsection, below.)

Three papers refer to ways in which participants in the quantified-self movement, or individuals who use smart sensors, can act on health data. The first paper mentions that one of the drives for data sharing is the quantified-self movement, which promotes and facilitates the sharing of data from wearables.^43^ The second paper mentions that movements such as “quantified self” use tracking devices, smart phones, and mHealth applications with the goal to evaluate their health status and enhance their personal health and performance capability.^26^ The third paper argues extensively that self-tracking devices provide users with a skewed quantified self that the user needs to actively interpret and harmonize with one’s own self-identity in order to translate health data into concrete options for self-management and preventions.^216^

Two papers discuss the effects of increased personal responsibility for one’s health on the good life that individuals can lead. The first paper says that the personalization of nutrition based on the individual’s biological characteristics might replace the ideal of the good life by “healthism” or otherwise raise excessive or narrowly focused expectations for individuals about their health.^180^ The second paper says that, because nanotechnological diagnostics may make individuals more responsible for their own health, healthcare politicians, and governments might hope that active patients will thereby ease economic constraints on the healthcare sector. It mentions that philosophers question what the effects of such shifts in the responsibilities of patients would be in terms of “good life ethics”. As examples of such changes, the paper mentions changes in morality regarding the value of “health” in cultural conceptions of what constitutes the good life, the motivation of individuals to act and interact in order to preserve or realize health, and the habits that individuals develop accordingly.^123^

On a more critical note, one paper argues that, while people are not responsible for their genetic profile, they may be perceived as responsible to some extent for their epigenetic and microbiome profiles. The paper argues that this could have discriminatory consequences for insurance and employment and reports that some direct-to-consumer microbiome test companies claim that the nondisclosure of test results by consumers could be considered fraud by insurance companies because consumers would fail to provide relevant information about individual risks to some diseases.^114^

Lastly, two papers discuss the actionability of omics information in particular domains. The first paper argues that there is a global discourse with moral outrage toward the rise in childhood obesity, which predominantly blames women for the intergenerational transmission of obesity. Against this discourse, the paper argues that an examination of epigenetic pathways calls into question the effectiveness of early-life obesity interventions that focus exclusively on the mother, which is important for the assessment of epigenetic responsibility.^217^ The second paper discusses the application of omics science to the study of mate choice and the ethical implications of so-called “pairomics”.^218^

#### Exposomic responsibility without direct individual control

To what extent should individuals be responsible for their exposome if many environmental exposures are not under their direct control? One paper reports that personalized medicine more and more relies on patients’ individual responsibility and that this runs the risk of lessened consideration of the social determinants of health.^206^ The issue of individual responsibility has been discussed a lot in the literature on epigenetics. One paper reports that many authors writing on the ethics of epigenetics point to a possible tension between collective and individual moral responsibility for epigenetic health.^91^ Similarly, another paper argues that the complexity of epigenetic programming of health highlights the inherent tension in the balance between individual responsibility for health and structural or societal responsibility for health.^110^ In the context of the epigenetic clock, one paper argues that the production of an objective and accurate surrogate marker for biological aging will reignite the discussion concerning how, and to what extent, individuals can be held accountable for their own behavior, and the impact that this has on theindividual’s health and the question how personal responsibilities can be balanced against the requirements of society (such as insurance and the provision of health care).^219^ Another paper argues that, although more evidence is required, epigenetic mechanisms are being implicated in the link between low socioeconomic status and poor health. On the one hand, it argues, knowledge about such types of epigenetic traits might allow us to move away from a genetic-deterministic perspective and empower individuals who have the opportunity to change their health status. The paper goes on to state that, on the other hand, this could lead to stigmatization and discrimination where individuals are deemed responsible for their health even if they are not in social situations where they are able to enact change that would alter their health status. The paper further discusses the role of the responsibilities of actors in genetic research, clinical practice, prenatal care, and the workplace.^220^ Taking an egalitarian approach to the issue, another paper argues that epidemiological research can suggest that health-related behaviors for which we are most tempted to blame individuals, such as smoking, do not require more attention from the perspective of individual moral responsibility for health, but from the perspective of whether the social structures that lead to health disparities are just.^221^ One paper takes an overview of this debate in epigenetics and argues that the literature on responsibility for one’s epigenome has focused too much on the limitations of individual responsibility, to the detriment of the role that moral luck plays in the grounds to dismiss the attribution of individual epigenetic responsibilities.^222^

### Occupational health and exposome research

The methods and results of exposome research can be applied to the field of occupational health. As in the case of biobanking, occupational health has its own developed literature on ethics. In this section, we present the ethical aspects that are relevant to occupational health from the perspective of exposome research that we found in the literature.

Three papers discuss the usage of biomarker tests for occupational health generally. The first paper claims that the use of susceptibility biomarkers should not result in discrimination or reduction of job opportunities for workers involved in research.^223^ Relatedly, the second paper argues that biomarker tests in occupational health may correlate with racial or cultural characteristics, which can further burden groups that already face discrimination. On the other hand, the paper argues, people might have the false assurance that workers that “pass” such tests constitute a hardier group that can be placed in the most hazardous jobs or can handle relaxed controls.^86^ The third paper warns that the exclusion of susceptible workers via preventative biomonitoring might lead to a false sense of safety that leads to potentially slackened hygienic measures. It also claims that such a preventive approach unjustly discriminates against susceptible individuals who would not have contracted disease from exposure.^224^

One paper discusses the obligations of employers and employees when using biomarker tests. It notes that workers may also have a responsibility to disclose results to insurers or potential employers, whereas workers with excess frequencies of various markers may put an ethical obligation on employers to provide follow-up monitoring. The paper also argues that the intentional or inadvertent disclosure of biomarker findings could have a chilling effect on a worker’s ability to get or keep jobs or health insurance.^86^

Two papers discuss why occupational health should not have a singular focus on biomarkers (this issue is related to the content of the section “Molecular redefinition of diseases”, below). The first paper discusses potential uses of biomarkers in the context of occupational and environmental epidemiology. It argues that an important consideration is that molecular techniques might cause us to redirect our focus from identifying risks in the exogenous environment to identifying high-risk individuals. The paper argues that this would direct our focus from public health epidemiology to a form of clinical evaluation and could distract from the important public health goal of creating a less hazardous environment.^181^ (See also the sections “Distinction participant–patient and epidemiology–medicine” and “Clinical translation of exposome research”.) The second paper argues that we need to resist the idea that only biomarker information is useful information about workers, as we should still pay attention to workers’ social, cultural, and political milieus, as well as what they say.^86^

Related to the above discussion, three papers discuss the distinction between occupational health and medical health. The first paper discusses the use of medical surveillance to satisfy the need for a graded response to environmental risks for occupationally attributable disease.^225^ The second paper argues that the right to refuse biomedical surveillance and medical intervention is an individual right that conflicts with majority rights. The paper explores the ethical implications of this conflict for occupational health and safety.^226^ Partly in response to the former two papers, the third paper argues that we should separate the ideas of biological monitoring, medical screening, medical surveillance, and environmental monitoring because ethical issues and medical controversies over their utility arise from a misunderstanding of what medical surveillance is and how it should be applied.^227^ (See also the sections “Distinction participant–patient and epidemiology–medicine” and “Clinical translation of exposome research”.)

Two papers discuss the relationship between advances that exposome research wishes to make (such as a deeper understanding of the biological responses to exposures) and occupational health. The first paper mentions that the development and characterization of the exposome, when integrated with the genome, may make it possible to: address all the factors that affect the health of the workforce, and better control work-related factors.^228^ The second paper argues that, because most occupational exposure limits are established on the basis of a relationship between a metric of external exposure and some toxicity endpoint, the human organism is considered to be a black box with only two ends of the relationship from exposure to outcome to be considered. But to establish a biological reference value, the paper argues, we need an understanding of the kinetics of the substance and the various factors influencing it, ie, the black box must be opened.^224^

Two papers discuss the ethics of occupational health more generally. The first paper provides an overview of ethical issues related to the use of biomonitoring for occupational health and argues that thorough cost–benefit analyses need to take place in cases of conflicts.^229^ The second paper sets out a number of principles for the use of biomonitoring and describes their ethical aspects, such as informed consent and the confidentiality of data.^230^

Four papers present and/or provide a short discussion of what they regard as pressing ethical aspects of occupational health. The first paper argues that the successful implementation of personalized medicine, omics technologies, and systems biology into occupational health settings requires addressing ethical, legal, social, and political considerations. It proceeds to discusses a number of these issues, such as equity, informed consent, and costs.^231^ The second paper argues that, although much has been written about the ethical aspects of using biological markers in occupational health research, the following issues are of importance for future considerations: inappropriate discriminatory effects on workers from employer usage of biomarkers related to behavior, personality, neurophysiologic characteristics, and epigenetic influences, and concerns from a social justice perspective that relate to investing scarce occupational and public health resources in specimen collection and analysis when other uses of funds may be of equal or more value.^228^ The third paper discusses what they regard as some of the most relevant ethical issues faced by those involved in biomonitoring for occupational health risk assessment: study planning, informed consent, confidentiality, communication, and susceptibility.^232^ The fourth paper discusses the value of biomonitoring for occupational safety and health, and provides an overview of key considerations for using biomonitoring for occupational health interventions.^233^

Lastly, two papers comment on the value of interviews for understanding the ethical aspects of occupational health. The first paper uses focus group interviews and an Internet discussion forum to understand how occupational health stakeholders represent ethical concerns raised by the use of biomarkers of exposure, effects, and susceptibility to harmful agents.^234^ The second paper reports on interviews with precision medicine research “thought leaders” on risks in precision medicine research and argues that these results have implications for research ethics.^235^

### Forensic science and exposome research

In theme #1, we mentioned that exposome research is aimed at improving health through an understanding of the exposome. However, this is not the only perspective that one might have on the exposome, as one could also investigate the exposome with the aim of advancing forensic science. For example, if there are certain situational or location-specific exposures that cause stable biological responses in the body, then discovering those responses in the internal bodily chemistry of a person could be a sign of that person having been in a particular situation or location in the past. From a systems-biological perspective, because exposome research fosters the analysis of multiple -omes at once, it could also allow forensic scientists to potentially create new holistic tests that provide more utility than tests that only use the information of a single -ome. In this respect, privacy protection assessments and forensic applications cover different sides of the same coin. Both need to understand the extent to which the exposome contains personal information. The former needs it to protect privacy, the latter needs it for the pursuit of criminal justice. In this paragraph, we present the ethical aspects that we found in the literature which relate exposome research to forensic science.

In the context of personal information, one paper argues that epigenetic tests may provide sensitive information about lifestyle, such as a person’s smoke exposure history. The paper says that this raises the question what types of epigenetic information about suspected criminals or immigrants seeking asylum forensic investigators and immigration control officers should be allowed to use. The paper goes on to state that this also raises the subsequent question of how much weight, if any, this type of evidence should be given in courts of law or administrative processes, given the error margins and confounding factors in most epigenetic tests such as age estimators.^201^ In the context of epigenetics, another paper mentions that there is an inherent conflict of interests between forensic issues and legal privacy regulations, which behooves forensic practitioners to be at the forefront in understanding and addressing complex ethical issues with potentially high stakes for the society and the individual.^236^ Relatedly, one paper argues that, with the advent of epigenomic tests in forensics, techniques to predict an unknown individual’s physiology (such as facial traits) based on their genotype can be expected to improve rapidly, eg, if genomic data related to facial traits is combined with epigenomic data related to chronological or biological age to improve facial portraits.^122^

Four papers report on the utility of a specific -ome for forensic purposes. The first paper reports that proteomic analysis of hair and plasma samples can be used to distinguish individuals and gather biogeographical information, such as ethnic background.^20^ Relatedly, the second paper argues that, because certain observations suggest that individuals might be uniquely and stably identified within a population based on their resident microbiota, the degree to which the human microbiome is identifiable is relevant to forensic genetics.^136^ The third paper reports that microbiomics tests are considered for forensic uses to gather more information about suspected criminals or victims, such as phenotypic or sociodemographic information.^122^ In that context, the fourth paper reports that skin-associated bacteria recovered from surfaces of computer keyboards and mice could be used as microbial “finger-prints” to identify individuals, which might be a valuable resource for forensic identification. It argues that, although the human microbiome is subject to modification by lifestyle and environmental changes, which can raise questions about the practical utility of such “finger-prints” after a certain amount of time, this does not preclude the possibility of combining an individual’s microbial data with genetic and other types of information to reveal personal and sensitive information.^60^

Omics technologies have also been used to create tests that estimate a person’s age. Such tests are often called “clocks”. Four papers comment specifically on the epigenetic clock, which is a test used to estimate age based on DNA methylation levels. The first paper argues that, in theory, the epigenetic clock could help make the basis of decisions about whether someone is lying about their age objective and transparent. It argues that such decisions need to be made in the context of immigration, forensic work, and sports. The paper claims that researchers should not develop, or make claims for, age-determining test without extreme care and wide discussion.^237^ The second paper extensively discusses ethical and legal considerations in forensic age estimation. It argues that DNA methylation biomarkers for age estimation may reveal a broader range of health-related information about the sample source, such as post-traumatic stress disorder and cognition strength measures. The paper mentions that one can argue that, in the context of unknown samples, extracting any information that could help identify the source should be considered beneficial. However, the paper mentions that such information could collide with the privacy of the source sample, which is an issue that the authors analyze and call more attention to.^238^ The third paper argues that a comprehensive framework for the ethical, legal, and social implications of DNA methylation clocks still needs to be formulated, even though they are already being used in forensics.^219^ Relatedly, the fourth paper argues that a human rights framework should guide further discussions about the nonmedical uses of epigenetic clocks. The paper presents and discusses potential ethical, legal, and social implications of nonmedical uses of epigenetic clocks. Implications mentioned by the authors are: the tension between actuarial and moral fairness, the promotion of free and informed consent, data governance and the protection of privacy and confidentiality, equity and nondiscrimination principles, identification and surveillance, the moral liability of criminals, scientific validity of commercialized epigenetic tests, and adequate interpretation and accuracy of findings and test results.^239^

We found two papers that comment on the use of biobank information in the murder case of Swedish foreign minister Anna Lindh. The first paper argues that using this information violated the consent taken from the legal representatives of children who agreed to donate blood for medical research purposes only. The paper mentions that this caused public distrust, as many Swedish citizens decided to withdraw from the biobank due to this event. The authors argue that this case and other similar cases pose the ethical question whether it would be justified to hand over samples that belong to biobanks and/or genetic newborns banks for forensic purposes, even without the consent of the donors or their legal representatives.^112^ The second paper argues that one reason why abiding by laws does not guarantee good research ethics is that legislation can change or be overruled. It argues that this could be seen when the murderer of Swedish foreign minister Anna Lindh was confirmed via a Swedish biobank that had blood samples for all newborn babies, even though such usage went against the Swedish Biobank Law in 2003.^18^

### Molecular redefinition of diseases

Because exposome research uses high-throughput methods and (multi-)omics technologies to measure biological responses to exposures, it aids in the increased understanding of health and disease on the molecular level. Due to the fact that our conception of health and disease is an important part of the basis on which we decide to act on health information, an increased “molecularized” understanding of health and disease is a fundamental issue that can have various downstream ethical implications.

This phenomenon is described in different ways by different papers. One paper argues that omics science and technologies allow for the analysis of the complex, longitudinal, and dynamic nature of the biological networks that fundamentally govern human health and disease (the italics are ours).^9^ Another paper mentions that it has been argued that part of creating a new taxonomy of human disease based on molecular biology involves describing and defining diseases based on their intrinsic biology in addition to traditional physical signs and symptoms.^32^ One paper reports that, unlike other illnesses such as congestive heart failure or sepsis, it is the case that mental illness or behavioral health concerns are not directly diagnosed via objective measures, laboratory reports, or other quantitative biomarkers, although recent trends suggest that this may change.^84^ Another paper argues that, within the framework of network medicine, omics technologies can help create reclassifications of disease that more precisely reflect pathogenesis and guide preventive, diagnostic, and therapeutic strategies. The authors anticipate that this will be followed by renewed disease phenotyping, improved prognostic information based on genomic and proteomic data, longitudinal studies of disease subtypes, and more effective and tailored treatments.^177^ In the context of epigenetics, one paper mentions that knowledge about health and diseases is being created and disseminated using increasingly more molecular language and molecular modes of thinking, and warns that doing so might obscure the nonmolecular economic and social context. The paper argues that such molecularization poses significant challenges to a balanced approach toward the management of health and disease.^176^

Several papers relate this phenomenon more directly to ethical considerations. One paper discusses how nanotechnology tends to redefine disease in molecular terms as deviations in molecular processes in the body. It argues that doing so grows the separation between the subjective experience of “illness” and the objectively diagnosed “disease”. The paper says that this process of “scientization” may cause patients to distrust their personal experience and make it more difficult to distinguish the boundary between what is normal and what is pathological. It goes on to argue that this phenomenon can also be seen as a step towards treating and transforming “the self itself” through medical biotechnology, which raises questions of (biopolitical) power and subjectivity.^123^ Another paper mentions that the precision medicine movement aims to redefine disease in terms of particular biomarker signatures, which can improve the overall risk/benefit profile of research and thus support the principle of beneficence. It argues that the results of research will be more applicable to the relevant clinical population and that biomarker-negative patients can be spared the burdens of ineffective treatments.^240^ One paper argues that the increased molecularization of disease could provide medical professionals with such a wealth of data that is not clear what results mean and when they justify action. The paper argues that there is also a risk of not having enough of the right data to comprehensively determine an individual’s state of health and need for an intervention.^123^ Relatedly, one paper claims that the integration of multiomic and lifestyle data has the potential pitfall of creating hazards due to the dehumanization of healthcare data.^66^ Another paper argues that, although there may be unintended consequences from translating social disparities into biological inequalities, a positive advantage of doing so is understanding the impact of these inequities on health before and after interventions, which can make epigenetic biomarkers an important part of future medical care.^110^ Lastly, one paper reports on discussions in epigenetics on the “molecularization of biography and milieu”—which is the embodiment of a person’s experiences and surroundings through the long-term biochemical changes in a person’s body that are caused by environmental and sociocultural circumstances. It argues that such biochemical changes can be epigenetic markers that are proof of past exposure.^91^

### Miscellaneous philosophical aspects

Several papers discuss the consequences of science for philosophy, or the consequences of philosophy for science. Here, we have grouped the arguments that refer to such causal interactions, which can subsequently affect the ethical aspects of exposome research.

One paper says that systems-biology and its applications have important ethical implications, such as identification, privacy, discrimination, integrity of life, and commercialization, but also prompts unanswered questions, such as whether in silico tests have the same legal status as therapies developed in vivo, what our cultural understanding of life is, and what the socioethical issues surrounding biological modification are.^241^ Another paper provides an ethical and epistemological analysis of the idea of “person” in personalized medicine.^206^

Two papers relate epigenetics to issues in philosophy. The first paper discusses the effects of the so-called “epigenetic turn” for one’s view of human nature.^242^ The second paper discusses the value of epigenetics for a theory of identity and subsequent issues such as moral responsibility, decision maintenance and advance directives, and value attribution to human developmental stages.^243^

One paper argues that research on human biomonitoring for biomarkers needs to be both objective and subjective: objective for determining the rationale of, designing and implementing the research, and subjective in the sense that concerns from the vantage point of subjects and other interested sectors of society are addressed, and to provide recommendations for preventive, remedial, or clinical action.^87^ (See also the section “Exposomic and genomic responsibility”.)

## Discussion

To the best of our knowledge, this article is the first comprehensive systematic review of the ethical aspects of exposome research. This review provides a descriptive overview that enables further research to perform ethical analyses that take into account what has been said in the literature and shows that the exposome research program ranges over a wide range of values. More specifically, exposome research touches upon well-known debates such as the ethics of biobanks, privacy, informed consent, and the return of results to participants. Many of the ethical aspects that we have described are parts of broader and existing debates in research ethics and bioethics. We think that exposome research affects these and other existing debates in essentially three ways. Because the exposome research program is stimulating the gathering of system-biological data (1) and the creation of big data tools (2) for ever-higher resolution analyses of health and disease (3), exposome research intensifies the effects of these three aspects of scientific progress on ethical debates.

Although we have not included genomics in our search query, many of the ethical aspects that are relevant to genomics research have an equivalent in exposome research. Such parallels can easily be drawn due to the fact that the very term “exposome” is modeled on the genome and that research program leaders are modeling exposome research on the “big science” of the Human Genome Project. However, our mapping of the ethical aspects of exposome research does not take an examination of parallels between genomics and exposomics as its analytic starting point. Doing so would have meant that our investigation into the ethical aspects that are relevant to exposome research would have provided unequal attention to other relevant parallels, such as those between exposome research and biomonitoring or epigenomics. Instead, we have examined the literature while having our eye out for any ethical aspect that might be relevant to exposome research. For the purposes of this review, we have used our knowledge of the ethics of genomics as an informative contrast that helps us to identify relevant ethical aspects of exposome research. The most explicit case in which genomics serves as such a contrast can be seen in the section on exposome counseling. The general absence of the ethics of genomics in this review should not be taken as a sign that work on that topic is not relevant for exposome research. Indeed, there are many valid and important parallels between the ethics of exposome research and the ethics of genomics. For example, both fields generate similar concerns with regard to debates on privacy protection, personal responsibility, and the return of incidental findings. Therefore, it is important for future normative analyses of the ethics of exposome research to take into account ethical arguments that have been made in related debates within the ethics of genomics.^244–246^

During our review, we have been asking ourselves whether there are any novel ethical debates or aspects that arise due to exposome research. Because we have performed our thematic analysis mainly by drawing from the approaches and fields that underly exposome research, our literature query precludes an answer to this question that arises from the existing exposome literature. However, using our knowledge of exposome research, we have grouped ethical aspects from underlying research fields and approaches that are relevant for ethical reflection on exposome tools, such as expotypes and a reference exposome. Thereby, we provide material for (novel) discussions of the ethical aspects of these tools as such. Furthermore, in the process of performing the thematic analysis for this review, we have discovered what we believe to be an ethical aspect that has not been explicitly thematized in the ethics literature, namely, research program ethics. We have attempted to incorporate this aspect into this review mainly via the formulation of the first theme on the goals of the exposome research program. Research program ethics concerns the code of values required for the successful formulation and execution of a research program. In the case of the exposome research program, the exposome concept itself has been coined to fulfil a scientific need for methodological improvement in exposure assessment, the advancement of the discovery of mechanisms by which the body responds to exposures, and to unite fragmented epidemiological research that focuses on particular categories of exposures instead of on all exposures.^1–3,247^ Concurrently, researchers have been setting up specific exposome research projects to execute on the advances that they wish to make with the investigation of the exposome.^85,248–252^ To answer the question how ethical theory can aid the exposome research program qua program, we need to think not just about the considerations mentioned in theme #1, but also about how to apply moral virtues such as creativity, integrity, and independence.^253,254^

Relatedly, it is also important to note that many of the arguments and claims that have been gathered in this review arise from various (often conflicting) perspectives on ethics. This means that there is work that needs to be done with respect to the integration of various (conflicting) considerations that are relevant to exposome research. Because the exposome research program has not fully matured yet, this means that there is an opportunity for both exposome and ethics researchers to propose and implement ethical considerations into exposome research.^4^

As mentioned in the methodological section of this review, we do not assess the quality of the various claims made in the literature and restrict ourselves to the systematic collection and description of ethical aspects in the sections above. Let us add that neither the length of a section nor the amount of references is an indication of how ethically pressing or important an issue is. The results of this review should not be interpreted as a “pie chart”, ie, a representation of what the most important ethical aspects of exposome research are. However, in light of both the issue of quality assessment and the large volume of ethical aspects in this review, we would like to emphasize a number of variables that are relevant for future work that draws upon the material that we have gathered. The first variable concerns technological progress. Some of the ethical aspects mentioned discuss problems that arise due to obsolescent or obsolete (bio)technology, whereas other problems have been or can be currently solved via new (bio)technological advances. Future analyses should therefore take stock of contemporary (bio)technology and assess whether or not the ethical aspects mentioned are still valid or could become relevant again. Second, some ethical aspects anticipate on future developments in exposome research. For example, neither the reference exposome nor exposome-based forensic tests are (fully) developed and used in practice. Because such technologies are still in the pipeline and not all (key) design choices have been made, there is still an opportunity to research their ethical aspects in parallel to their development and subsequently steer the way in which values are incorporated into these technologies.^4^ Third, it is important to note that not all of the ethical aspects mentioned in this review are legitimate ethical considerations from the perspective of all ethical theories. For example, there are many critics of distributive justice, who, correspondingly, would have a different perspective on the actionability of health inequalities. In other words, researchers should think critically about the legitimacy of the various ethical aspects that we have found in the literature.

We wish to highlight three fundamental aspects of exposome research that we believe would benefit most from further ethical reflection.

The first aspect concerns the action-implications of the knowledge that is generated by exposome research. What is the nature of the external validity of the findings, data and (statistical) tools of exposome research? Is there something general that we can say about the external validity of exposome knowledge, or is exposome knowledge highly context dependent? Correspondingly, is there general guidance that can be given for individuals, policymakers, (eHealth) companies, and clinicians for the optimal use of such findings, data, and (statistical) tools for improving health? Because many of these tools and data are still being designed and generated, there is an opportunity here as well for explicit reflection and subsequent integration of values in these technologies before such technologies are embedded and hard to change.^4^ Reflections on this question will have downstream consequences for various topics mentioned in this review, such as research standards, biobank sustainability, and nearly all topics in theme #5.

The second aspect relates to the position of exposome research vis-à-vis environmental epidemiology and medicine. As mentioned by papers in the sections “Distinction participant–patient and epidemiology–medicine” and “Clinical translation of exposome research”, there are differences between epidemiology and medicine with respect to the way in which they generate and interpret data, conceptualize study subjects as participants or patients, the way in which interventions should take place if study results are actionable for the health of study subjects, and whether or not there is (or should be) a doctor–patient relationship present for participants. As exposome research matures, there is a need to determine where exposome research stands on the environmental epidemiology—medicine axis, and correspondingly, which norms from each field are applicable or need to be reconceptualized. Importantly, this second aspect revolves around the occupational identity of “the exposome researcher”— which was touched upon in the section “Institutional policies and educational standards”. What are the competencies of exposome researchers, and to what extent are epidemiological, medical, or clinical–epidemiological norms applicable to the work that they do? Such questions are especially pertinent for principal investigators of exposome research projects, as they are most likely to be in the position to determine what is required to become a (future) exposome researcher.

The third aspect pertains to meaning and ethical implications of bias. During the writing of this review, we discovered that various papers from different disciplines use the term “bias” in mutually exclusive ways. In the section “Bias in data, analysis, algorithms, and artificial intelligence”, we have attempted to disambiguate the term “bias” to the extent that doing so was required to categorize the ethical aspects that we found on the topic. The literature generally considers bias to be a problem, or at least an important phenomenon that needs to be handled correctly. But we know of no general perspective on the meaning, purpose, and value of the term “bias” that untangles the mutually exclusive uses of the term that we found, and also shows a clear way to evaluate different types of bias. In this respect, the problem of understanding what bias is in general, and the question how we should evaluate different biases, is a so-called “wicked problem” that requires untangling.^4^ To avoid ambiguous use of the term “bias” and subsequent confusion about how to act on bias, future research would benefit from a univocal understanding of what bias is and how to act on it.

### Limitations

This systematic review provides a comprehensive overview of the ethical aspects of exposome research as mentioned in articles that can be found in the academic literature. The ethical aspects that are collated in this article were included after a wide screening of the scientific literature on exposome research and related research fields and approaches that come together in exposome research.^8^ However, this review has three main limitations. First of all, since we have constructed a query that draws on research fields and approaches that underly exposome research, we have had to exercise our own judgment with respect to the research fields and approaches that underly exposome research. Even though we have drawn upon literature that shows how certain research fields and approaches come together in exposome research, there is a degree of latitude here, and other researchers could have made different choices (reporting bias). Relatedly, the way in which we have selected and grouped ethical aspects, under single aspects as well as thematically, is necessarily affected by our own philosophical frameworks. This means that, also for this aspect of our review, other researchers would have probably made different selections and groupings (reporting bias). Third, because not all ethical aspects discovered or discussed by researchers are necessarily included by them in their publications, this review should not be taken to necessarily cover all ethical aspects that researchers know about (arguably a form of publication bias).^255^

## Conclusion

Research on the human exposome has a very broad range of possible applications in fields such as public, occupational and environmental health, eHealth, and medicine. Despite the fact that the various sub-fields and disciplines that encompass exposome research often have a much more developed ethical discourse, this review provides the first comprehensive overview of ethical aspects that are relevant to exposome research as a whole. Our overview provides a stepping-stone for further discussions about the ethics of exposome research because it gathers relevant moral considerations from the literature itself. Because the tools and products of exposome research are still in the design phase, there is an opportunity for exposome researchers and ethicists to proactively assess ethical challenges before the technology becomes embedded and difficult to change. Our article can be viewed as a call for exposome researchers to think about ways in which their research program can be improved with the guidance that ethics can provide. As we have emphasized via our categorizations and discussion, ethical reflection can help exposome researchers with their own aims, ie, with improving our knowledge of health and disease and devising ways of acting on those advances.

## Supplementary Material

Executive summary

Supplemental file I
