# Supplementary material for: The ethical aspects of exposome research: a systematic review
Source: Exposome. Author manuscript; Available in PMC 2023 Sep 22. (PMC7615114; doi:10.1093/exposome/osad004)
Supplement: Executive summary [file EMS181732-supplement-Executive_summary.docx]

# The Ethical Aspects of Exposome Research: An Executive Summary

Exposome research draws on research fields and approaches that have originally yielded ethical debate and is rapidly developing. However, there is little ethical reflection on exposome research as such. Therefore, it is pertinent that we map and investigate the ethical issues that are relevant for exposome research. Because exposome research has not matured yet and much of its tools are still in the design phase, there is an opportunity for exposome researchers and ethicists to explicitly think about the way in which values are incorporated into exposome research. In our article, we provide a comprehensive overview of the ethical aspects of exposome research via a systematic review that categorizes the various ethical aspects that are mentioned in the exposome research literature and the literature of approaches and fields that underly exposome research. We identified five major themes and highlight three topics in need of further ethical reflection.

## Theme 1: Research program goals

The exposome research program aims to improve human health by creating the tools necessary to analyze all bodily exposures and their corresponding biological responses throughout an individual’s lifespan. This prompts the question how the goals of the exposome research program align with other goals, such as advancing human wellbeing or alleviating human suffering. It also raises the question which general considerations should be incorporated into the concrete goals of specific research projects, such as policy goals or ‘stakeholder’ voices.

## Theme 2: Research standards

Exposome research and other multi-omics research create new tools, perform complex interdisciplinary analyses and result in scientific uncertainty. In light of these advances, there is a need for academic journals, educational programs, ethics committees and researchers using new measurement technologies to adequately update their research standards. Doing so would safeguard a multitude of values, such as research integrity, innovation and researcher competency.

## Theme 3: Research tools

Exposome research uses data-driven agnostic screening approaches to discover and track expotypes (clusters of exposures). Such data gathering might conflict with the principle of data minimization. Relatedly, data sharing and integration are of enormous value to exposome research for many reasons, such as generating more scientific progress, allowing for data verification & replication and creating a reproducible and accountable scientific practice. However, data sharing and integration practices need to be evaluated in light of values with which they might conflict, such as privacy, participant integrity and data security. Next, because population health research requires the statistical study of a subset of individuals from a population, there are also ethical aspects relating to statistical bias. This includes ideas such as the need for a diverse dataset to achieve external validity, the potential of artificial intelligence to reinforce existing misdiagnoses and attempts to distinguish between desirable and undesirable biases. In terms of research infrastructure, biobanks are an important source of data for exposome research. It is mentioned that biobanks need to be economically sustainable and require standardized and harmonized policies that do not hamper innovation. Intellectual property rights are also a recurring topic in the literature; discussions contain familiar questions with respect to the justification of biological patents, as well as worries about the chance that patents hamper research progress. Because researchers are in the process of creating a reference exposome, we grouped discussions about the value of population-level data for individual risk assessment with discussions on the reference epigenome.

## Theme 4: Study participants

Exposome research analyzes an increasing number of exposures and biomarkers within populations, which allows for an increased resolution of both disease etiology and individual behavior within populations. In this respect, exposome research touches on all aspects of the rights of study participants and the obligations of researchers towards study participants. For example, there are questions concerning the property rights that participants hold and can exercise over their bodily materials and data, and the extent to which participants should be burdened and rewarded for participating in research. Similarly, the increased complexity, scope and resolution of exposome research requires a reevaluation of the ways in which researchers can best safeguard (data) privacy, present potential participants with good informed consent forms, as well as determine which type of consent is best for their research. Researchers also need to think about the communication of results to study participants: which data they return, how to return them, and how or whether to provide exposure reduction strategies and exposome counseling. Important aspects include the participant’s ‘right to know’ and the question whether reporting results either causes unnecessary fears or empowers participants. Similar discussions occur on the topics of privacy and informed consent, which contain questions surrounding the best ways in which to protect privacy and organize informed consent; especially in light of increased scientific knowledge, advances in digital infrastructures and rights of participants.

## Theme 5: Consequences of products

As its research program comes to fruition, the products of exposome research will have consequences to various domains of human activity. To anticipate the ethical aspects of those consequences, several papers suggest employing methods such as SWOT-matrices, speculative scenario’s, comparisons to the ethics of genomics, and ethics parallel research. Because exposome research aids in the push towards more precise and personalized health knowledge, it also has several effects on public health practices and the use of reference values. Furthermore, there are discussions of how exposome research can affect ‘distributive justice’ in either creating or reducing unjustified health inequalities. Similarly, there are discussions on how exposome research could be used to discriminatory ends. The potential effects of exposome research on law and international treaties are also discussed in the literature. Advances in research might provide new types of knowledge or evidence that require updating environmental and reproductive tort law, human rights and ethics declarations, and non-discrimination and privacy law. Next, because exposome research could allow for better stratification and diagnosis, there are discussions about ethical aspects related to the clinical translation of exposome research, such as: the proper treatment of patients who are not eligible for personalized treatments, the explainability of artificial intelligence in diagnosis, and direct-to-consumer diagnostic testing. There are also discussions on how individuals can benefit from exposome information and whether exposome research will lead to more individual responsibility for health issues that individuals can’t directly affect. Other fields of exposome-application are occupational health and forensic science, where (classic) issues of privacy, discrimination and responsibility arise. Exposome research also aids in an increased molecular understanding of health and disease. This raises ethical aspects relating to data overload for medical professionals, the increasing distinction between molecular problems and felt symptoms, and the corresponding potential neglect of non-molecular factors relating to health.

## An Exposome Ethics Research Agenda: three topics

In light of the specific ethical aspects that we have found, we wish to highlight three fundamental aspects of exposome research that we believe would benefit most from further ethical reflection.

First, what is the nature of the external validity of the findings, data, and (statistical) tools of exposome research, and what corresponding general guidance can be given for individuals, policy makers, (eHealth) companies and clinicians for the optimal use of such findings, data and (statistical) tools for improving health? An answer to these questions will have downstream consequences for many of the topics mentioned in our review, such as research standards, biobank sustainability, and nearly all values in theme #5.

Second, where does exposome research stand on the distinction between environmental epidemiology and medicine? Correspondingly, which norms from each field are applicable, or perhaps need to be reconceptualized, when it comes to topics such as study participation, informed consent, privacy and the communication of results?

Third, we found many ambiguous and mutually-exclusive usages of the term ‘bias’ in the literature. Both from a statistical and ethical perspective, it is important to consider and act on bias. In order to avoid confusion on this topic, future research would therefore benefit from a univocal understanding of what bias is and how to act on it.
