# Supplementary material for: The ethical aspects of exposome research: a systematic review
Source: Exposome. Author manuscript; Available in PMC 2023 Sep 22. (PMC7615114; doi:10.1093/exposome/osad004)
Supplement: Supplemental file I [file EMS181732-supplement-Supplemental_file_I.docx]

Supplemental file I

Caspar W. Safarlou*^1^, Karin R. Jongsma^1^, Roel Vermeulen^1,2^, Annelien L. Bredenoord^1,3^

1: Department of Global Public Health and Bioethics, Julius Center for Health Sciences and Primary Care, University Medical Center Utrecht, Utrecht, The Netherlands

2: Department of Population Health Sciences, Utrecht University, Utrecht, The Netherlands

3: Erasmus School of Philosophy, Erasmus University Rotterdam, Rotterdam, The Netherlands

Manuscript title: The Ethical Aspects of Exposome Research: A Systematic Review

Mailing address of corresponding author: C.W. Safarlou, internal mail no. STR 6.131, Universiteitsweg 100, 3584 CG Utrecht, The Netherlands

Email address of corresponding author: c.w.safarlou@umcutrecht.nl

Exposome and derivatives only:

- PubMed:
  - (exposome[mesh] OR exposom*[tiab]) AND (ethics[mesh] OR morals[mesh] OR ethics[subheading] OR ethic*[tiab] OR moral*[tiab] OR bioethic*[tiab] OR bio-ethic*[tiab] OR elsi[tiab] OR elsa[tiab]) AND (dutch[Filter] OR english[Filter])
- Embase:
  - ('exposomics'/exp OR 'exposome'/exp OR 'exposom*':ti,ab,kw) AND ('morality'/exp OR 'ethics'/exp OR 'ethic*':ti,ab,kw OR 'moral*':ti,ab,kw OR 'bioethic*':ti,ab,kw OR 'bio-ethic*':ti,ab,kw OR 'elsi':ti,ab,kw OR 'elsa':ti,ab,kw) AND (english:la OR dutch:la) AND [embase]/lim NOT 'conference abstract'/it
- Web of Science:
  - (TS=(exposom*)) AND (SU=("medical ethics") OR WC=(ethics OR "medical ethics") OR TS=(ethic* OR moral* OR bioethic* OR bio-ethic* OR elsi OR elsa)) AND LANGUAGE: (English OR Dutch)

Exposome, derivatives and underlying research fields and approaches:

- PubMed:
  - (exposome[mesh] OR metabolomics[mesh] OR metabolome[mesh] OR proteomics[mesh] OR proteome[mesh] OR transcriptome[mesh] OR epigenomics[mesh] OR epigenome[mesh] OR biomonitoring[mesh] OR exposom*[tiab] OR metabolom*[tiab] OR proteom*[tiab] OR transcriptom*[tiab] OR epigenom*[tiab] OR omic*[tiab] OR biomonitoring[tiab] OR bio-monitoring[tiab] OR "biological monitoring"[tiab] OR ((biomarkers[mesh] OR biomarker*[tiab] OR bio-marker*[tiab] OR "biological marker"[tiab] OR "biological markers"[tiab]) AND ("precision medicine"[mesh] OR "precision medicine"[tiab] OR "personalized medicine"[tiab] OR "personalised medicine"[tiab]))) AND (ethics[mesh] OR morals[mesh] OR ethics[subheading] OR ethic*[tiab] OR moral*[tiab] OR bioethic*[tiab] OR bio-ethic*[tiab] OR elsi[tiab] OR elsa[tiab]) AND (dutch[Filter] OR english[Filter])
- Embase:
  - ('exposomics'/exp OR 'exposome'/exp OR 'metabolomics'/exp OR 'metabolome'/exp OR 'proteomics'/exp OR 'proteome'/exp OR 'transcriptomics'/exp OR 'transcriptome'/exp OR 'epigenome'/exp OR 'multiomics'/exp OR 'biological monitoring'/exp OR 'exposom*':ti,ab,kw OR 'metabolom*':ti,ab,kw OR 'proteom*':ti,ab,kw OR 'transcriptom*':ti,ab,kw OR 'epigenom*':ti,ab,kw OR 'omic*':ti,ab,kw OR 'biomonitoring':ti,ab,kw OR ‘bio-monitoring’:ti,ab,kw OR 'biological monitoring':ti,ab,kw OR (('biological marker'/exp OR 'biomarker*':ti,ab,kw OR ‘bio-marker*’:ti,ab,kw OR ‘biological marker*’:ti,ab,kw) AND ('precision medicine':ti,ab,kw OR 'personalized medicine'/exp OR 'personalized medicine':ti,ab,kw OR 'personalised medicine':ti,ab,kw))) AND ('morality'/exp OR 'ethics'/exp OR 'ethic*':ti,ab,kw OR 'moral*':ti,ab,kw OR 'bioethic*':ti,ab,kw OR 'bio-ethic*':ti,ab,kw OR 'elsi':ti,ab,kw OR 'elsa':ti,ab,kw) AND (english:la OR dutch:la) AND [embase]/lim NOT 'conference abstract'/it
- Web of Science:
  - (TS=(exposom* OR metabolom* OR proteom* OR transcriptom* OR epigenom* OR omic* OR biomonitoring OR bio-monitoring OR "biological monitoring" OR ((biomarker* OR bio-marker* OR “biological marker” OR “biological markers”) AND ("precision medicine" OR "personalized medicine" OR “personalised medicine”)))) AND (SU=("medical ethics") OR WC=(ethics OR "medical ethics") OR TS=(ethic* OR moral* OR bioethic* OR bio-ethic* OR elsi OR elsa)) AND LANGUAGE: (English OR Dutch)
